# ﻿Evolutionary relationships of Fish Lake Valley Tui Chub *Siphateles obesus* ssp. (Teleostei, Cypriniformes, Leuciscidae) and a new genus of leuciscid minnows from the Alvord Basin, western United States

**DOI:** 10.3897/zookeys.1261.151636

**Published:** 2025-11-21

**Authors:** Matthew A. Campbell, Serra C. Perry, Khyana N. Yearwood, Grace Auringer, Nick Buckmaster, Amanda J. Finger

**Affiliations:** 1 Genomic Variation Laboratory, Department of Animal Science, University of California, One Shields Avenue, Davis CA, 95616, USA University of California Davis United States of America; 2 Fishes and Marine Invertebrates, University of Alaska Museum of the North, 1962 Yukon Drive, Fairbanks AK, 99775, USA University of Alaska Museum of the North Fairbanks United States of America; 3 California Department of Fish and Wildlife, Inland Deserts Region, Heritage and Wild Trout Program, Bishop, CA 93514, USA California Department of Fish and Wildlife, Inland Deserts Region Bishop United States of America

**Keywords:** ASAP, *
Epizon
*, Great Basin fishes, molecular phylogenetics, phylogenetic networks, species delimitation methods

## Abstract

The Tui Chubs, *Siphateles* spp., are found widely across the Great Basin and in some adjacent regions. Nearly all diversity of *Siphateles* has been consolidated under the name *S. bicolor* and there are numerous isolated populations of Tui Chubs of uncertain taxonomic standing and therefore unclear conservation priority. The Fish Lake Valley Tui Chub (FLVTC) has been recognized informally as *S. bicolor* ssp. 4 with a limited natural distribution in Fish Lake Valley in southwest Nevada. Considering that a rigorous examination of the phylogenetic relationships of the FLVTC and other Tui Chubs has not been conducted, the FLVTC is placed in a taxonomic framework by first applying a species delimitation method to *S. bicolor* sensu lato using mitochondrial data and then conducting phylogenetic analyses of genome-wide SNP data. *Siphateles bicolor* is better characterized by seven species, all with existing names, which here are considered to be valid *Siphateles* species. Furthermore, the separation of Alvord Basin *Siphateles* from other *Siphateles* is apparent as a deeply divergent lineage. As a result, we propose *Epizon* Campbell & Finger, **gen. nov.** to contain these fishes. The Fish Lake Valley Tui Chub is found to be the earliest-branching lineage of *S. obesus* in our SNP data set and are highly differentiated from other *S. obesus*. These findings are concordant with geologic evidence that indicates that Fish Lake Valley became connected to the broader Lahontan Basin ~2 million years ago, with gene flow possible until ~0.5 million years ago. Based on the geographic distribution and magnitude of genetic divergence, we find the recognition of FLVTC as a subspecies of *S. obesus* is appropriate.

## ﻿Introduction

Encompassing a vast area of western North America, the Great Basin is generally bordered by the Columbia River to the north, the Sierra Nevada to the west, and by the Rocky Mountains to the east (Houghton 1976). The name Great Basin refers to the fact it is endorheic, and it is composed of large sub-basins, the Lake Bonneville System, the Central Basins, the Death Valley System, the Northwest Lakes Basin, and the Lake Lahontan System (Houghton 1976). The ichthyofauna of the Great Basin is taxonomically diverse, but not consistently distributed across all sub-basins and limited in species diversity.

Aquatic species diversity in a biogeographic region is largely a function of historical processes that generate and preserve species diversity. For fishes in the Great Basin, two of these processes may be dispersal into and subsequent speciation within the Great Basin. Processes that generate diversity are opposed by processes that reduce aquatic species diversity, such as long periods of habitat reduction, aridity, and isolation. Isolation may also contribute to species diversity if populations are not reconnected or, if they do, reproductive isolation is in place. In the Great Basin, the Pleistocene was characterized by dramatic changes in habitat, with large pluvial lakes appearing and disappearing (Mifflin and Wheat 1979), alternating periods of dispersal and high habitat availability with periods of isolation, differentiation, and extinction. These periods of high and low water availability operated over drainage patterns that have been shaped on million-year time scales (e.g., Minckley 1986). During the history of the Great Basin, ecological and habitat preferences of fishes also influenced dispersal and population connectivity. High-gradient systems in foothills and mountains were subject to a higher frequency of inter-drainage headwater stream capture in comparison to lacustrine spillover affecting low-gradient systems. These higher gradient systems are typically occupied with mountain suckers (*Pantosteus* spp.), salmonids (*Oncorhynchus* spp. and *Prosopium* spp.) and sculpins (*Cottus* spp.). The low gradient and low elevation habitats more typically are occupied by suckers (*Catostomus* spp. and *Chasmistes* spp.), minnows (Leuciscidae), pupfishes (*Cyprinodon* spp.), and salmonids with this habitat preference (Smith et al. 2002). The distribution and diversity of Great Basin fish species result from these habitat associations and shared historical processes as well as chance. Nonetheless, the evolutionary history of all Great Basin fishes is reflected in their genetic structuring and genetic diversity (e.g., Finger et al. 2022; Su et al. 2022; Campbell et al. 2023).

While species diversity is generated, maintained, and reduced by natural processes, its recognition is heavily influenced by scientific effort (Lundberg et al. 2000). Great Basin fishes remain incompletely documented and characterized taxonomically, and number ~55 recognized species-level taxa. Over such a large area, the Great Basin contains about 8% (55/683) of recognized fish species diversity in North America (Jenkins et al. 2015). These are from the Gasterosteidae (*n* = 1), Cottidae (*n* = 3–4), Cyprinodontidae (*n* = 5–7), Goodeidae (*n* = 4), Salmonidae (*n* ~ 10), Catostomidae (*n* ~ 13) and Leuciscidae (*n* ~ 16) (Smith et al. 2002; Sigler and Sigler 2014; Unmack et al. 2014; Campbell et al. 2023; Harris et al. 2025).

The leuciscid minnow lineages have a long presence in the Great Basin fossil record despite the challenges associated with preservation and recovery of fossils from these fishes, and several species are still present in this system. Fossil leuciscid minnows are known from at least the middle Miocene in the Great Basin (~16 mya) (Cavender 1986). Three genera have been persistent and are found widely, these are *Gila* Baird & Girard, 1853, the senior synonym of *Moapa* Hubbs & Miller, 1948, *Rhinichthys* Agassiz, 1849, and *Siphateles* Cope, 1883. *Siphateles* is known to be well-represented in the fossil record in the Miocene and Pleistocene of the Lahontan Basin and Mojave River (Smith et al. 2002). The genus *Siphateles*, despite being widely distributed across the Great Basin, contains only three valid species (Fricke et al. 2025). *Siphateles bicolor* (Girard, 1856) is distributed broadly across phylogeographic regions, including the Great Basin, in the Klamath River drainage, the Sacramento River drainage (Pit River), and the Columbia River drainage. The other two species are closely-related and are found in the Alvord Basin: *S. alvordensis* (Hubbs & Miller, 1972) and *S. boraxobius* (Williams & Bond, 1980), and they may belong to a separate genus altogether (Schonhuth et al. 2012). Divergence time estimation indicates that the Alvord Basin leuciscids has been an independent lineage for ~10 million years (Rabosky et al. 2018). All other Great Basin *Siphateles* have been placed under *S. bicolor* (Girard, 1856) and within *S. bicolor* there are various distinctive and isolated fish populations, but they are of uncertain taxonomic standing and thus unclear conservation priority. Molecular dating places the age of the lineage of fish classified as *S. bicolor* at ~ 12 million years (Kumar et al. 2017; Rabosky et al. 2018). In conjunction with fossil evidence, the distribution and ages of these fishes indicates that there was ample time and space for diversification within *Siphateles* beyond the currently recognized three species. Other genera with distributions spanning the Great Basin of that age exhibit distinct species such as *Rhinichthys* (Moyle et al. 2023), *Pantosteus* Cope, 1875 (Unmack et al. 2014), *Catostomus* Lesueur, 1817, and *Chasmistes* Jordan, 1878 (e.g., Campbell et al. 2023). In particular, there are geographically and genetically structured populations within these genera distributed among Columbian, Sacramento/Klamath and Lahontan geographic regions that may be recognized as species.

Based on the age, wide range, occurrence across phylogeographic regions known for endemism, and lower elevation habitat preference that promotes fewer drainage basin exchanges it is plausible that *S. bicolor* contains several species and subspecies-level taxa. Lack of recognition of these taxa is, in part, because a rigorous and comprehensive phylogenetic examination of Great Basin *Siphateles* has not been undertaken to test the species boundaries of these diverse populations. An understanding of the diversity within *S. bicolor* and the relationships between this lineage and others will inform management and conservation decisions in this arid landscape.

Here we conduct this analysis of Great Basin *Siphateles* and focus on the Fish Lake Valley Tui Chub (FLVTC) which has been recognized informally as *S. bicolor* ssp. 4. It occupies a limited natural distribution in Fish Lake Valley in southwest Nevada where it is the only extant native fish. Specifically, we conduct a molecular phylogenetic study with the aim of identifying the evolutionary relationships of FLVTC, current composition of the lineage, and its taxonomic standing.

## ﻿Materials and methods

### ﻿Mitochondrial phylogeny and species delimitation

We obtained representative sequences of *Siphateles* from the NCBI GenBank drawing from a mitochondrial cytochrome b (*cytb*) PopSet 28190045 (Harris 2000). Outgroup sequences were selected from 12 leuciscid minnows in the Laviniinae subfamily with *Chrosomus erythrogaster* (Rafinesque, 1820) used for rooting (Schonhuth et al. 2012). Additional *Siphateles* sequences with identifying information that could expand representation were obtained from GenBank through blastn searches against the NCBI Nucleotide database using *cytb* sequences from PopSet 28190045 (Altschul et al. 1997). During the collection of public sequence data, we obtained a single potential representative mtDNA sequence from the Owens Valley (AF370056.1). The Owens Valley is known to contain distinctive endemic *Siphateles* lineages, but also out-of-basin genetics from human mediated movements (Chen et al. 2007). The single publicly available sequence was ambiguous due to these factors and we undertook Sanger sequencing of *cytb* sequences from 12 individuals from the Owens Valley of known native backgrounds to represent endemic *Siphateles* mitochondrial lineages. We used two primer sets to amplify two fragments of the *cytb* gene that were combined into a longer sequence. The primer sets were previously reported by Zardoya and Doadrio (1998) and used to generate previously published data from *Siphateles* (e.g., Schonhuth et al. 2012). The primers used were Glu-F 5’-GAAGAACCACCGTTGTTATTCAA-3’; Cytb-R 5’-TCTTTATATGAGAARTANGGGTG-3’, Cytb-F 5’-CACGARACRGGRTCNAAYAA-3’ and Thr-R 5’-CCTCCRATCTYCGGATTACA-3’ (Zardoya and Doadrio 1998).

Polymerase Chain Reactions (PCR) were carried out in a total volume of 25 µL, using 5x Colorless GoTaq® Flexi Buffer (5 uL per reaction) with a final concentration of 200 µM of dNTP, 2 mM MgCl2, 0.2 µM of each primer, 0.625 U of GoTaq® DNA polymerase, and 15 ng of DNA extract. After an initial denaturation step of 2 min at 95 °C, we ran 40 PCR cycles consisting of 1 min at 95 °C, 1 min at 45 °C, and 1 min and 45 s at 72 °C. These cycles were followed by a 7-min final extension step at 72 °C. Forward and reverse direction chromatograms were aligned with Geneious (https://www.geneious.com), and then the two primer set contigs together were mapped to a reference sequence from NCBI (*Siphateles bicolor*; accession: AY096010.1) with the ‘Map to Reference’ function within Geneious.

A multiple sequence alignment (MSA) of *cytb* sequences was made with MAFFT (Katoh et al. 2002; Katoh and Toh 2008; Katoh and Standley 2013) and Maximum-Likelihood (ML) phylogeny inferred with IQ-TREE2 (Nguyen et al. 2014; Minh et al. 2020). Model selection was conducted by specifying ModelFinder Plus (-m MFP) (Kalyaanamoorthy et al. 2017) and support for nodes evaluated with 10,000 rapid bootstraps (-bb 10000) (Hoang et al. 2018).

To delimit possible species within *S. bicolor*, mitochondrial sequences identified as *S. bicolor* were uploaded to the Assemble Species by Automatic Partitioning (ASAP) webserver (Puillandre et al. 2021, https://bioinfo.mnhn.fr/abi/public/asap/asapweb.html). Simple, uncorrected distance was specified p̂.

### ﻿Individual and genomic sampling

**Figure 1.**
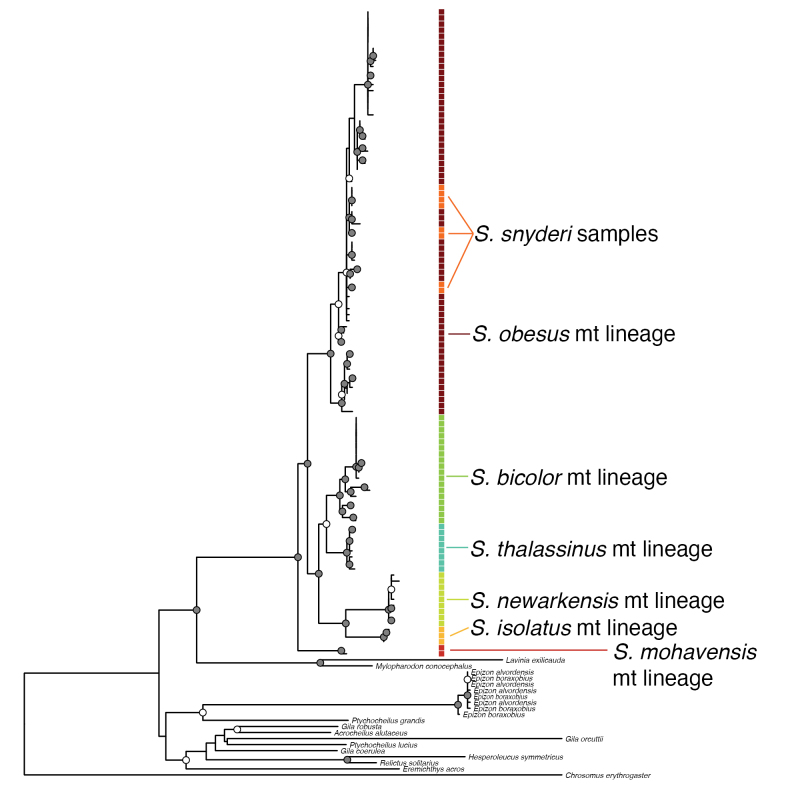
Maximum Likelihood phylogeny of mitochondrial (mt) cytochrome b (*cytb*) data featuring ‘*Siphateles bicolor*’ labeled sequences with results of species delimitation indicated for six clusters. Nodal support is indicated by gray circles for bootstrap support (BS) > 90%, white circles for 90% > BS > 75% and not indicated for BS < 75%.

**Figure 2.**
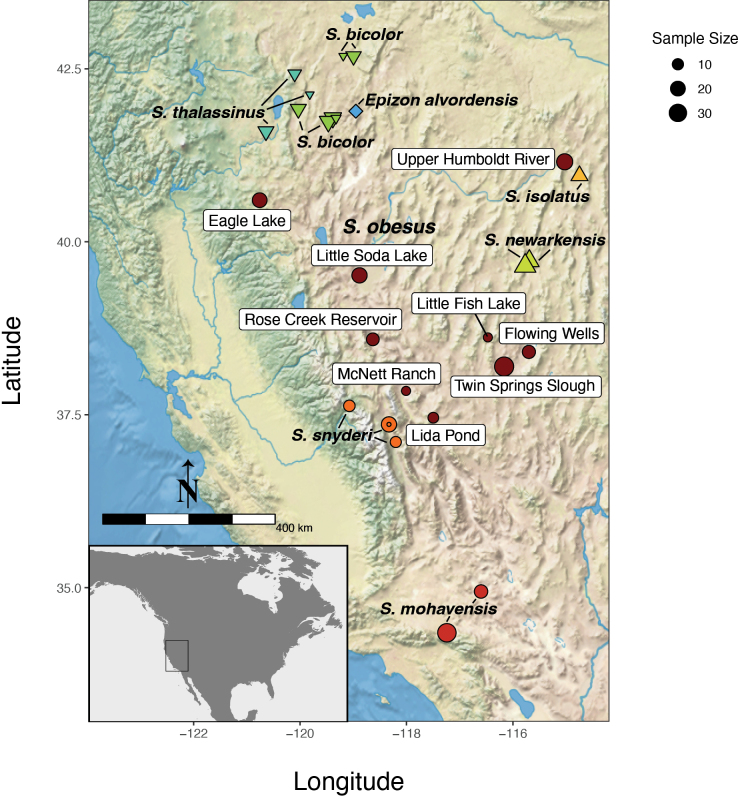
Sampling map of *Siphateles* spp. sequenced in this study with RADseq included in analyses (*n* = 427). The outgroup taxa of Arroyo Chub (*Gila orcuttii*, *n* = 2) and Sacramento Splittail (*Pogonichthys macrolepidotus*, *n* = 9) are not shown. Specific collection localities for *S. obesus* are labeled. The inset indicates the extent of the main map within North America.

Access to *Siphateles* tissues was possible through recently collected specimens or from tissue archives. Material from across the Great Basin range of the genus (Fig. 2, Table 1), representing known divergent lineages of *Siphateles* with emphasis on potential near relatives of Fish Lake Valley Tui Chub was targeted (Fig. 1, Harris 2000). Fin clips were either dried and stored in coin envelopes or in 95% ethanol prior to extraction of DNA. Sampling included representatives of *S. alvordensis* (*n* = 13, one location) and a variety of potential species-level lineages currently classified as subspecies under *S. bicolor*: *S. mohavensis* Snyder, 1918 (*n* = 74, two locations); *S. isolatus* (Hubbs & Miller, 1972) (*n* = 25, one location); *S. newarkensis* (Hubbs & Miller, 1972) (*n* = 25, two locations); *S. bicolor* (*n* = 74, six locations); *S. thalassinus* (Cope, 1883) (*n* = 43, three locations); *S. snyderi* (Miller, 1973), including ‘toikona’ lineage fish (Chen et al. 2007) (*n* = 66, four locations and *n* = 50, two locations respectively). *Siphateles obesus* (Girard, 1856) were considered potential near relatives of FLVTC and nine locations were sequenced (*n* = 223). One location of *S. obesus* is located in Fish Lake Valley (McNett Ranch) and was considered representative of the FLVTC lineage. Sequences for rooting phylogenetic analyses were obtained from *Gila orcuttii* (Eigenmann & Eigenmann, 1890) and *Pogonicthys macrolepidotus* (Ayres, 1854).

**Table 1.** Taxonomic and geographic sampling. The larger geographic area (Vicinity) is indicated along with each sampling location. The sample size (*n*) used in analyses with the median read count after filtering is given as well as the total number of individuals sequenced (Total *n*). For the year collected, if collected in different years, the number retained from each year for analyses is given. The latitude and longitude and source of samples are also provided with any additional pertinent information. The taxonomic sampling is from *Pogonichthys macrolepidotus* (Ayres, 1854), *Gila orcuttii* (Eigenmann & Eigenmann, 1890), *Epizon alvordensis* (Hubbs and Miller, 1972) and *Siphateles* Cope, 1883. *Siphateles* is abbreviated *S.* in the taxon column with *S. thalassinus* (Cope, 1883), *S. bicolor* (Girard, 1856), *S. newarkensis* (Hubbs & Miller, 1972), *S. isolatus* (Hubbs & Miller, 1972), *S. snyderi* (Miller, 1973), *S. mohavensis* Snyder, 1918, and *S. obesus* (Girard, 1856).

Taxon	Vicinity	Sampling location	Median read count	*n*	Total *n*	Year collected	Latitude, Longitude	Source
*Pogonichthys macrolepidotus*	Unknown	Unknown	2461487	9	12			CDFW Archives
*Gila orcuttii*	Mojave River Drainage	Desert Discovery Pond	2053059	2	2	2012		
*Epizon alvordensis*	Sheldon Wildlife Refuge	Thousand Creek Gorge	7589069	10	13	2023	41.8873, -118.9520	
*S. thalassinus*	Pit River	Big Sage Reservoir	4944824	12	12	2005	41.5957, -120.6405	
*S. thalassinus*	Valley Falls	Honey Creek	2065216	8	15	2011	42.4262, -120.1009	
*S. thalassinus*	Warner Spring	Twenty Mile Slough	1439984	3	16	2004	42.1291, -119.8177	
*S. thalassinus*	Modoc County	Cowhead Slough	2964294	12	20	2023	41.9193, -120.0322	
*S. bicolor*	Catlow Valley	Rock Creek	1136559	3	6	2011	42.6854, -119.1888	
*S. bicolor*	Harney County	Kueny Canyon	3307963	11	12	2005	42.6818, -118.9958	
*S. bicolor*	Sheldon Wildlife Refuge	Andy’s Place	2348208	15	16	*n* = 4, 2022 *n* = 11, 2023	41.7916, -119.3846	
*S. bicolor*	Sheldon Wildlife Refuge	Bitner Ranch	3167076	16	16	*n* = 4, 2022 *n* = 12, 2023	41.7368, -119.4686	
*S. bicolor*	Sheldon Wildlife Refuge	Horse Canyon	7898712	4	4	2022	41.7863, -119.3244	
*S. newarkensis*	Newark Valley	NN4	3110134	20	25	2022	39.7227, -115.6919	
*S. newarkensis*	Newark Valley	NV12	5185390	25	25	2022	39.6498, -115.7700	
*S. isolatus*	Independence Valley	Warm Springs	3322395	14	25	2022	40.9545, -114.7490	
*S. snyderi*	Mammoth Mountain	Sotcher Lake	1571551	11	23	*n* = 1, 1998 *n* = 10, 2022	37.6286, -119.0737	
*S. snyderi* ‘toikona’	Owens River	Cottonwood Pond (White MT Research Center)	1787193	21	25	2010	37.3606, -118.3276	
*S. snyderi* ‘toikona’	Owens River	Mule Spring	1410489	10	25	2010	37.1061, -118.3276	
*S. snyderi*	Owens River	NE Pond (White Mountain Research Center)	2154310	22	25	2017	37.3606, -118.3293	
*S. snyderi*	Owens River	SE Pond (White Mountain Research Center)	2307926	2	10	2022	37.3604, -118.3294	
*S. snyderi*	Owens River	SW Pond (White Mountain Research Center)	2532879	2	8	2022	37.3604, -118.3296	
*S. mohavensis*	Mojave River	Camp Cady	2230368	16	19	*n* = 6, 1997 *n* = 10, 2005	34.9451, -116.5993	
*S. mohavensis*	Mojave River Drainage	Tui Slough	1662314	35	55	2011	34.3483, -117.2411	
*S. obesus*	Carson River, Carson Desert	Little Soda Lake	2696470	22	25	2006	39.5147, -118.8833	
*S. obesus*	Fish Lake Valley	Lida Pond	2590847	9	28	2022	37.4571, -117.4959	
*S. obesus*	Fish Lake Valley	McNett Ranch	2008366	6	25	2021	37.8443, -118.0086	
*S. obesus*	Hot Creek Valley	Twin Springs Slough	3127174	38	49	*n* = 16, 2011 *n* = 22, 2022	38.1969, -116.1656	
*S. obesus*	Humboldt River System	Upper Humboldt River	2920540	25	25	2022	41.1564, -115.0271	
*S. obesus*	Lassen County	Eagle Lake	6658527	20	20	2023	40.6015, -120.7589	
*S. obesus*	Little Fish Lake Valley	Little Fish Lake	1754730	6	12	2011	38.6181, -116.4710	
*S. obesus*	Railroad Valley	Flowing Wells	1647665	15	23	*n* = 14, 2011 *n* = 1, 2022	38.4081, -115.7003	
*S. obesus*	Walker Lake	Rose Creek Reservoir	3571471	14	16	2023	38.5899, -118.6329	

Determination of *Siphateles* lineages was in part informed by mitochondrial phylogenetic results (Suppl. material 1: fig S1), inclusion in previous studies, and by geographic locations. *Siphateles alvordensis* samples are from the Alvord Lake basin, and not from Borax Lake and can represent *S. alvordensis*. The location, Thousand Creek Gorge was investigated previously in genetic study of numerous Alvord Lake basin *Siphateles* populations by Smith et al. (2019) and is representative of *S. alvordensis* in that study. *Siphateles mohavensis* is extinct from its type locality, was translocated for conservation, and we sampled the descendants of this management action (Chen et al. 2013). *Siphateles isolatus* is also extinct from its type locality, but was sampled from nearby locations in Independence Valley. *Siphateles newarkensis* is endemic to springs in the Newark Valley with a restricted distribution, described from a spring on the western side of the valley. The NV12 sampling location is located ~5.5 km from the likely type locality as indicated by Hubbs and Miller (1972) as springs on an alluvial fan near Diamond Peak, on the west side of Newark Valley (Harris 2000). *Siphateles bicolor* is described from Upper Klamath Lake, with phylogenetic affinities to the material examined in this study indicated as *S. bicolor*, with Rock Creek in Catlow Valley sequenced for both mitochondrial and nuclear data types (Suppl. material 1: fig S1). *Siphateles thalassinus* is described from Goose Lake, in Oregon and we did not obtain material from that location for this study. However, we obtained material from close geographically proximity from the Warner Valley and the Pit River (Goose Lake may be considered part of the Pit River drainage). There are mtDNA lineages similar to Goose Lake samples present in Cowhead Slough (Suppl. material 1: fig S1) as well. Samples of *S. thalassinus* from Twenty Mile Slough and Big Sage Reservoir were previously analyzed by Chen et al. (2009) with associated museum specimens OS 17847 and OS 17853 and are representative of *S. thalassinus* in that study. *Siphateles snyderi* and *S. snyderi* ‘toikona’ tissues were obtained largely from refuge populations maintained for this taxon, and may be considered representative of this lineage. The Mule Spring sampling location has been previously investigated as a refuge population of the ‘toikona’ genetic lineage, founded by translocation from Cabin Bar Ranch in 1990 (Chen et al. 2007). *Siphateles obesus* was described from the Humboldt River, and we were able to sample from this drainage basin for this study, and the mitochondrial phylogenetic analysis and geographic distribution are concordant with recognizing the sampling as being representative of *S. obesus*.

Genomic DNA was extracted from the chub fin clips using the QIAGEN DNeasy Blood and Tissue Kit according to the manufacturer’s protocol. Because the fin clips used in this project could be of substantial age (25+ years, Table 1), we adjusted the elution step to increase DNA yields by incubating molecular grade water at 56 °C for 10 min. DNA was eluted in 25 μL of warmed molecular grade water then centrifuged for 4 min at 5788xg. This step was repeated in order to produce a total of 50 μL of final DNA product.

Library preparation followed the BestRAD protocol (Ali et al. 2016) with the *SbfI* restriction enzyme and individual barcodes ligated to sequences. Libraries were pooled and sequenced across two lanes on an NovaSeqX with paired-end 150 bp 25B sequencing chemistry. Sequence data were demultiplexed to the plate level then combined from different lanes for individual demultiplexing. Paired reads from individuals were aligned to the *G. orcuttii* reference genome (GCA_026230005.1) with the Burrows-Wheeler aligner and the MEM algorithm (bwa mem) (Li and Durbin 2010). Alignments were sorted, filtered for proper pairing, PCR duplicates removed, and number of aligned reads calculated with SAMtools (Li et al. 2009). Individuals with more than 1 million filtered and aligned reads were retained for analyses.

### ﻿Genome-wide phylogenetic analyses

A set of SNP genotypes was generated by first calling SNPs with Analysis of Next Generation Sequence Data (ANGSD) (Korneliussen et al. 2014). Quality control of SNPs was enforced by requiring SNPs to be present in 95% of individuals, minimum mapping and base quality values of 20 (-minMapQ 20, -minQ 20), a significance value of 1.0 x 10^-6^ (-SNP_pval 1e-6), a posterior probability value of 0.95 (-postCutoff 0.95) and a minimum Minor Allele Frequency of 0.01 (-minMaf 0.01). A SAMtools genotype calling model was used and a PLINK-formatted file generated. Subsequently, the PLINK-formatted file was converted to a VCF-formatted file with PLINK (Purcell et al. 2007) and the VCF-formatted file was filtered to a MAF of 0.05 and ‘pruned’ with BCFtools to obtain unlinked SNPs (Danecek et al. 2021). The specific options supplied for pruning with BCFtools were +prune -m 0.20 -w 10000. A Multiple Sequence Alignment (MSA) of SNPs in PHYLIP format was made with the vcf2phylip.py script (https://github.com/edgardomortiz/vcf2phylip/blob/master/vcf2phylip.py) from the pruned VCF file. Subsequent conversion of the PHYLIP-formatted file to NEXUS format was done with functions of the *ape* package in R (Paradis 2010).

A concatenated Maximum-Likelihood (ML) phylogenetic tree was generated at the individual level with IQ-TREE2 (Minh et al. 2020). A General Time Reversible (GTR) model of nucleotide evolution with ascertainment bias correction (+ASC) was specified. Support for nodes was evaluated with the ultrafast bootstrapping algorithm (-bb 1000 -nm 2000 -bcor 0.9) (Hoang et al. 2018). A species-tree that incorporates the multispecies coalescent (explicitly modeling incomplete lineage sorting) was constructed with SVDQuartets in PAUP* (Swofford 2003; Chifman and Kubatko 2014, 2015). Individuals were pooled at the collection location level for this analysis, with 10 million random quartets evaluated. Nodal support was evaluated with 1,000 bootstrap replicates. A third analysis, an implicit phylogenetic network was undertaken to visualize possible discordance more fully as a result of hybridization and incomplete lineage sorting with SplitsTree (Huson and Bryant 2005). Uncorrected distances were used with the Neighbor-Net algorithm.

## ﻿Results

### ﻿Mitochondrial phylogeny and species delimitation

We analyzed an alignment of 127 sequences with sequence accessions and metadata provided in Suppl. material 2. Mitochondrial sequences are from 12 leuciscid minnow species not classified in *Siphateles* (*n* = 12 sequences) and 115 of the sequences are indicated to be from different *Siphateles* species. These 115 sequences are split between four sequences identified as *S. alvordensis*, four as *S. boraxobius*, and the remainder (*n* = 107 sequences) as *S. bicolor*. We obtained seven new sequences from the Owens Valley with two sequences from ‘toikona’ fish. The alignment of all 127 sequences is 1,140 bases in length with 284 parsimony-informative sites.

The best-choice model under the Bayesian Informative Criterion (BIC) was GTR+F for inference of a ML phylogeny. *Siphateles* is indicated to be polyphyletic with Alvord Basin leuciscids more closely related to other fishes. The ASAP algorithm identified six clusters within *S. bicolor* labeled sequences with a threshold distance of 0.012 or five clusters with a threshold distance of 0.016. The six-cluster approach was preferred with an ASAP score 3.50 compared to 5.00 with a lower score considered better. The six-cluster result is presented as Fig. 1 with additional collection information and subspecific nomenclature presented as Suppl. material 1: fig S1 and indicates the following species may be recognized: *Siphateles mohavensis*, *S. isolatus*, *S. newarkensis*, *S. thalassinus*, *S. bicolor* and *S. obesus*. The five cluster result combines *S. thalassinus* and *S. bicolor*. *Siphateles snyderi* sequences are nested within *S. obesus*.

### ﻿Individual and genomic sampling

Sequences from a total of 427 *Siphateles* from 30 locations with more than 1 million filtered and aligned reads were retained for downstream analyses. Outgroups are represented by two *G. orcuttii* and nine *P. macrolepidotus* sequences.

### ﻿Genome-wide phylogenetic analyses

Genotype calling with ANGSD produced an initial 24,073 SNPs. After filtering for a minimum MAF 0.05, pruning with BCFtools and converting to a PHYLIP-formatted file, the MSA consists of 4,125 sites. These sites have 4,118 distinct alignment patterns, 2,497 parsimony informative sites, 152 singleton sites and 1,476 sites considered invariant by IQ-TREE2.

**Figure 3.**
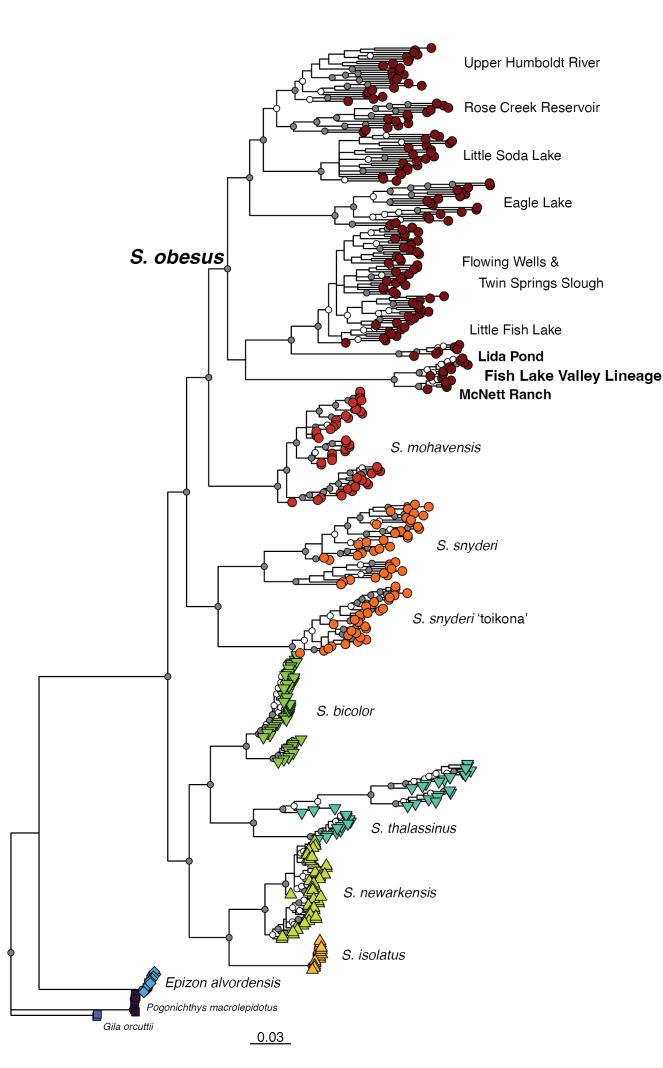
Maximum Likelihood phylogeny of *Siphateles* generated from 2,649 SNPs considered variable by IQ-TREE2. For *S. obesus*, sampling locations are indicated. Nodal support is indicated by gray circles for bootstrap support (BS) > 90%, white circles for 90% > BS > 75% and not indicated for BS < 75%.

The concatenated ML phylogeny is presented as Fig. 3 with tips individually labeled to collection locality as Suppl. material 1: fig S2. A sister relationship is present between *S. mohavensis* and *S. obesus*. Bootstrap Support (BS) for this arrangement is 97%. Support for monophyly of *S. mohavensis* is maximal, with support for the monophyly of *S. obesus* being high (BS = 93%). Within *S. obesus*, the monophyly of a Fish Lake Valley genetic lineage is maximal as well as the reciprocal monophyly of the McNett Ranch and Lida Pond sampling locations. The placement of the Fish Lake Valley genetic lineage as the sister lineage of a combined Little Fish Lake Valley and Railroad Valley fish receives only modest (BS = 73%) support. The monophyly of a broader Lahontan Basin grouping of *S. obesus* receives high (BS = 91% support).

**Figure 4.**
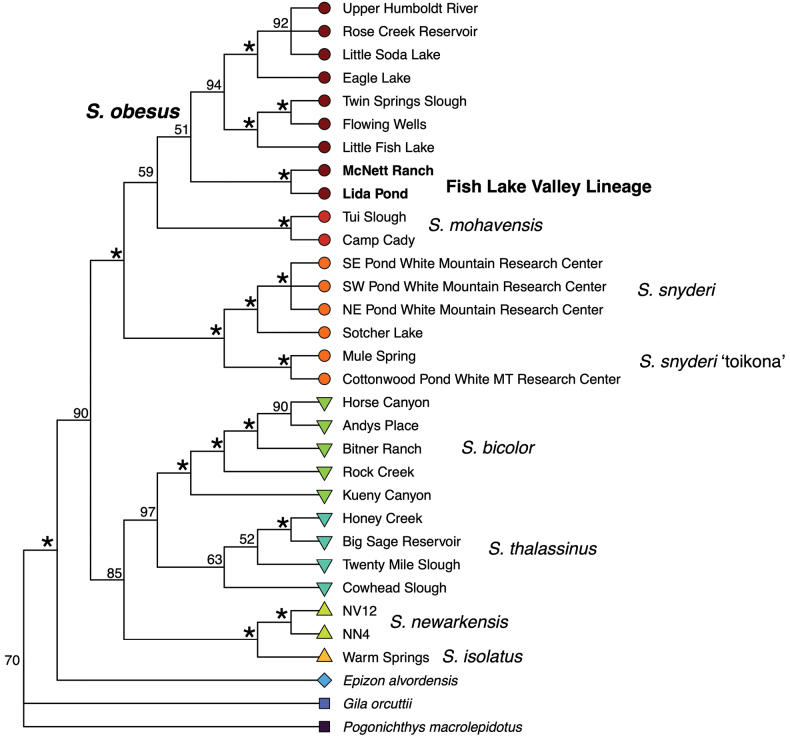
Species tree of *Siphateles* from SVDQuartets generated from 4,125 SNPs. Individuals from the same collection locations were pooled for this analysis. Nodal support values are from 1,000 bootstrap replicates. Bootstrap values of 100% are indicated by an asterisk and bootstrap values < 50% are collapsed into polytomies.

The species-tree analysis (Fig. 4) resolves *S. mohavensis* as the sister lineage of *S. obesus* with maximal support (BS = 100%), as well as the monophyly of these two lineages. Within *S. obesus*, the Fish Lake Valley genetic lineage is the earliest-branching and contains fish from McNett Ranch and Lida Pond. Subsequently, *S. obesus* is divided between a combined Railroad Valley and Little Fish Lake Valley lineage and a broader Lahontan Basin grouping.

**Figure 5.**
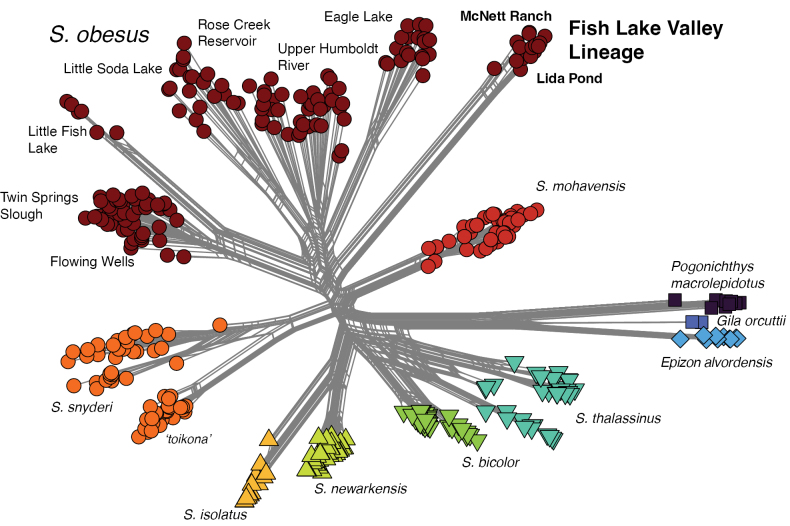
Implicit phylogenetic network of *Siphateles* from SplitsTree generated with the neighbor-net algorithm from 4,125 SNPs. Sampling locations within *Siphateles obesus* are indicated.

The phylogenetic network (Fig. 5) again shows a close relationship among the three species-level lineages of *S. snyderi*, *S. mohavensis*, and *S. obesus*. Within *S. obesus*, the separation into three main lineages - Fish Lake Valley (with two sampling locations, McNett Ranch and Lida Pond), Railroad Valley/Little Fish Lake Valley and a broader Lahontan Basin group is indicated.

### ﻿A new genus of leuciscid minnows from the Alvord Basin

We find Alvord Basin leuciscids are a deeply diverged lineage from all other fishes classified within *Siphateles* (Figs 1, 3–5). We propose a new genus to contain the Alvord Basin leuciscids, *Epizon* gen. nov., with two valid species *E. alvodensis* (Hubbs & Miller, 1972) and *E. boraxobius* (Williams & Bond, 1980).

#### ﻿ Epizon

Taxon classificationAnimaliaCypriniformesLeuciscidae

Campbell & Finger gen. nov.

B764B9D5-C249-5D36-916A-684708DECE76

https://zoobank.org/75BD3B60-1234-40A3-A2E5-016D63B7A119

##### Type species.

*Gila alvordensis* Hubbs & Miller, 1972.

##### Included species.

**Figure 6.**
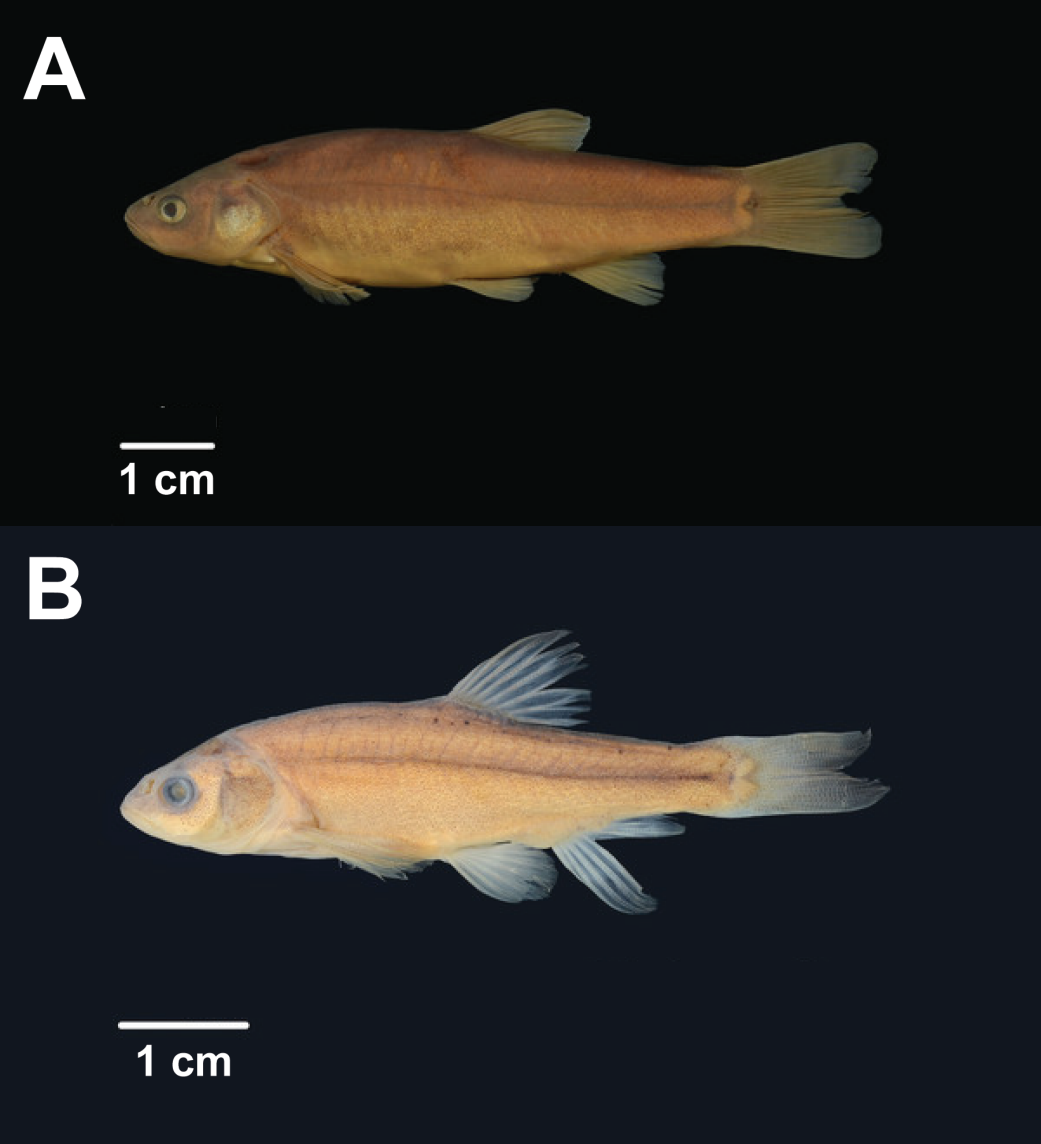
**A.** Holotype of *Epizon alvordensis* UMMZ 130495, female, 71 mm SL (Collected 26 July 1934 by the Hubbs family under field identifier M34-87); **B.** Holotype of *Epizon boraxobius* UMMZ 203329, male, 50 mm SL (Collected 5 August 1977 by K. Howe and colleagues under field identifier Z203329). Photographs provided by the University of Michigan Museum of Zoology.

*Gila alvordensis* Hubbs & Miller, 1972: Trout Creek, Alvord Desert, Harney County, Oregon, USA. The location given is “just below the canyon and just below bridge where roads to Denio, Jordan Valley, and Fields meet” (Hubbs and Miller 1972, pg. 102). Holotype: UMMZ 130495 (image examined, Fig. 6A). Paratypes: UMMZ 130496 (198, sharing same field identifier M34-87), UMMZ 130533 (536), UMMZ 130534 (112), UMMZ 130535 (299). Valid as *Epizon alvordensis*.

*Gila boraxobius* Williams & Bond, 1980: Borax Lake, Harney County, Oregon, USA. Holotype: UMMZ 203329 (image examined, Fig. 6B). Other specimens from the type locality: UMMZ 203330 (107), OS 4137 (12), OS 4138 (179), OS 12820 (3). Valid as *Epizon boraxobius*.

##### Diagnosis.

*Epizon* differs from *Siphateles* by having radii in all fields of the scale whereas radii are found only in the posterior field in *Siphateles*. Members of *Epizon* typically have seven dorsal- and anal-fin rays in contrast to the eight or more dorsal- and anal-fin rays in *Siphateles*. Scales are reduced in size and more embedded in *Epizon* in comparison to *Siphateles* with larger and less embedded scales. These attributes are discussed by Hubbs and Miller (1972), Miller (1973) and Williams and Bond (1980). *Epizon* is noted to exhibit sexual dimorphism with male fins longer than females, a characteristic not reported in *Siphateles* (Williams and Bond 1983; Williams et al. 1980; Baker et al. 2021; Sigler and Sigler 2014). *Epizon* may be diagnosed from *Siphateles* by 63 nucleotide substitutions within the mitochondrial cytochrome b gene (*cytb*), 46 of which are transitions and 17 of which are transversions (Table 2).

**Table 2.** Mutations identified within the mitochondrial cytochrome b (*cytb*) gene separating *Epizon* gen. nov. from *Siphateles* Cope, 1883 based on 115 total sequences of these taxa (*Epizon* gen. nov., *n* = 8 and *Siphateles*, *n* = 107). The position in the *cytb* sequence is given, the state observed in *Epizon* gen. nov., the state observed in *Siphateles*, and if the difference is transitional (Ti) or transversional (Tv).

Position	*Epizon* gen. nov.	* Siphateles *	Transition or Transversion
21	T	C	Ti
24	T	C	Ti
27	G	A	Ti
60	T	C	Ti
66	G	A	Ti
72	C	A or G	Tv
81	C	T	Ti
105	C	T	Ti
141	C	A	Tv
145	T	C	Ti
183	C	T	Ti
189	C	T	Ti
198	C	A or G	Tv
199	G	A	Ti
219	A	T	Tv
234	C	T	Ti
255	T	C	Ti
258	A	C	Tv
309	T	C	Ti
318	G	A	Ti
321	T	C	Ti
351	C	A or G	Tv
387	G	C	Tv
393	C	T	Ti
399	T	C	Ti
426	C	G	Tv
444	T	C	Ti
459	C	A or G	Tv
477	T	C	Ti
492	T	C	Ti
498	A	G	Ti
522	G	C or T	Tv
576	G	A	Ti
592	C	T	Ti
598	C	T	Ti
609	C	T	Ti
612	C	A or G	Tv
652	G	A	Ti
663	T	C	Ti
717	T	C	Ti
735	C	T	Ti
759	G	A	Ti
771	T	C	Ti
819	T	C	Ti
834	C	T	Ti
855	C	A or G	Tv
876	A	C	Tv
885	G	A	Ti
897	T	A or G	Tv
901	T	C	Ti
909	C	G	Tv
912	G	A	Ti
918	T	C	Ti
933	A	G	Ti
957	C	T	Ti
999	A	T	Tv
1002	T	C	Ti
1017	G	A	Ti
1023	A	G	Ti
1026	T	C	Ti
1059	A	C	Tv
1068	T	C	Ti
1122	C	T	Ti

##### Description, distribution, and ecology.

*Epizon* is a genus of small to medium-sized minnows in the family Leuciscidae (Fig. 6). Teeth pharyngeal, mouth terminal. The color is variable with habitat occupied, varying from nearly black dorsally with speckled golden sides and a silver belly in canyon habitats to light green dorsally without speckling laterally and a white belly in downstream habitats (Williams and Bond 1983). *Epizon boraxobius* tends to be moderately dark, olive-green dorsally, silver-colored laterally and ventrally (Williams and Bond 1980). Average number of dorsal-fin rays of 7, anal rays 7, pelvic rays 8, and caudal-fin rays 19. *Epizon alvordensis* commonly has 13 pectoral-fin rays and *E. boraxobius* 14 (Williams and Bond 1980).

*Epizon* is endemic to the Alvord Basin of Eastern Oregon of the Northwest Lakes subregion, of the Great Basin (Houghton 1976). The Alvord Basin is a north-south oriented valley bounded by Steens Mountain to the west and the Owyhee Plateau to the east, with formation of the valley during the Miocene (Orr and Orr 2012). It appears that the ancestral lineage of *Epizon* was isolated during this time. During the Pleistocene, the pluvial Lake Alvord was ~19 km wide and 113 km long during maximal extent (Reheis 1999). Subsequent drying isolated fishes in limited suitable habitats in a cyclical fashion, resulting in *E. alvordensis* being more broadly distributed within the basin of former Lake Alvord and *E. boraxobius* restricted to the thermal spring-fed Borax Lake and proximate habitats. Within Borax Lake, fish live typically for a year and are of a smaller size (33–45 mm SL) whereas in the diverse habitats elsewhere in Alvord Basin *E. alvordensis* fish live longer and grow larger, 4–5 years and ≤ 120 mm SL (Williams and Bond 1983; Sigler and Sigler 2014). A sample of 50 fish from Gridley Springs were reported to be 27–91 mm SL with a peak at 30-38 mm SL and fish distributed into larger sizes ≤ 91 mm SL (Williams and Bond 1983). As such, *E. boraxobius* had been noted prior to formal taxonomic description as a dwarf form of *E. alvordensis*. Borax Lake is fed by springs with 35–40 °C outflows resulting in a lake of typically 29–32 °C temperatures, with *E. boraxobius* avoiding temperatures above 34 °C and a loss of equilibrium at 34.5 °C (Williams and Bond 1983). *Epizon alvordensis* occupies a variety of habitats in springs, creeks, ponds, and reservoirs, in seven of eight historically occupied drainages (Scheerer et al. 2015). This taxon is found in cold, cool, and warm waters, but not above 31.1 °C (Williams and Bond 1983). Differentiation of the two species of *Epizon* may be driven by adaptation to the unique characteristics of the thermal-spring discharge with possible sympatric speciation of *E. alvordensis* and *E. boraxobius* (e.g., Smith et al. 2019). In addition to the characters of Williams and Bond (1980), nuclear genetic data separates the two species (Smith et al. 2019). Remple (2013) reports that larger eye diameter, longer snout length and longer head length differentiating *E. alvordensis* and *E. boraxobius* are apparent in early life stages and can be used to differentiate larvae.

Nuptial tubercles are present in males with sexual dimorphism reported in *E. boraxobius* based on relative length of fins, which are all longer in males than females (Williams and Bond 1980). This characteristic is also apparent in *E. alvordensis* (Sigler and Sigler 2014). Given the constant temperatures of Borax Lake, year-round spawning occurs with *E. boraxobius*. *Epizon alvordensis* in thermally fluctuating habitats, spawns only once a year (Williams and Bond 1983). *Epizon* are opportunistic omnivores with a diet closely related to the production of their habitats and variable with seasons (e.g., Williams and Williams 1980). Top dietary items are microcrustaceans, chironomids and diatoms (Williams and Bond 1983).

##### Etymology.

Romanized Greek version of επιζών, meaning survivor in reference to the persistence of this genus in the diverse and challenging desert habitats it has found itself. Gender masculine.

## ﻿Discussion

### ﻿Composition of *Siphateles*

Analyses presented here (Figs 1, 3–5) and by others (Schonhuth et al. 2012, 2014, 2018; Rabosky et al. 2018;) indicate that the Alvord Basin leuciscids are substantially diverged from other members of *Siphateles*. Upon description, the affinities of *Gila alvordensis* Hubbs & Miller, 1972 were to the then subgenus Siphateles. The recognition of *Siphateles* as a genus by Simons and Mayden (1998) did not include representation of the Alvord Basin leuciscids in their molecular data set; however, Alvord Basin leuciscids were included in the elevation of *Siphateles* at that point. Subsequent molecular phylogenetic analyses of Alvord Basin leuciscids lack clear resolution of their placement, neither being a sister lineage of *Siphateles* nor being more-closely related to other fishes convincingly indicated (e.g., Schonhuth et al. 2012, 2014, 2018). We advocate that the Alvord Basin leuciscids should be placed in a separate genus, Epizon gen. nov., described in the results section of this manuscript. We choose to recognize two valid species of *Epizon* although mitochondrial barcode data does not resolve these two species phylogenetically (Fig. 1). Previous investigation of *E. alvordensis* and *E. boraxobius* with microsatellite and genome-wide SNP data, however, find support for a phylogenetic division of these two taxa that predates the end of Pleistocene (Smith et al. 2019). The implication is that these two lineages may have speciated sympatrically within the Alvord Basin. These lines of evidence as well as anatomical divergence and adaptation to hot-springs habitat of *E. boraxobius* lead us to conclude in support of its validity.

Monophyly of a less inclusive *Siphateles* is supported across different molecular data types – mitochondrial loci, nuclear loci, and genome-wide SNP data (e.g., Schonhuth et al. 2012, 2014, 2018) and Figs 1, 3, 4 in this study. Mitochondrial, genome-wide SNP data and microsatellite data indicate that this less inclusive *Siphateles* may be divided into several allopatric units representing distinct clades (e.g., Figs 1–3; Harris 2000; Remple 2013). *Siphateles* as defined here, contains seven species: *S. isolatus*, *S. newarkensis*, *S. bicolor*, *S. thalassinus*, *S. mohavensis*, *S. snyderi* and *S. obesus*.

A major phylogenetic grouping present within *Siphateles* is of *S. isolatus* and *S. newarkensis*, found in Independence Valley and Newark Valley, Nevada respectively. These lineages are found in the Central Basins region of the Great Basin following Houghton (1976). Within these species, the subspecies *S. isolatus euchila* (Hubbs & Miller, 1972) from Fish Creek Valley as well as the nominal subspecies *S. isolatus isolatus* from Independence Valley may be reasonably recognized. Further studies specifically on this question, including more comprehensive anatomical investigation are needed to evaluate the taxonomic status of *S. isolatus isolatus* and *S. isolatus euchila*.

Another major phylogenetic grouping is of *S. bicolor* and *S. thalassinus*. Overall, these lineages are found distributed in the Klamath and Pit river basins, the Northwest Lakes section of the Great Basin (Houghton 1976), and the Columbia River basin more generally (e.g., Lubinski and Scholz 2021). *Siphateles bicolor* contains several subspecies including the named *S. bicolor columbianus* (Synder, 1908) and *S. bicolor eurysoma* (Williams & Bond, 1981). Unlike Harris (2000), sequences from the ‘Silver Lake’ sampling location are nested within *S. bicolor* (Suppl. material 1: fig S1) and because of that we do not conclude it represents an undescribed distinct species as indicated by Harris (2000). We also find *Siphateles thalassinus* to be valid with the subspecies *Gila bicolor vaccaceps* Bills & Bond, 1980 placed in molecular phylogenetic analysis as a subspecies of *S. thalassinus*. That is, *S. thalassinus vaccaceps* (Bills & Bond, 1980), found in the Cow Head Basin.

A third grouping of *Siphateles*, including *S. mohavensis*, *S. snyderi* and *S. obesus* is also found in our genome-wide analyses. Geographically, these lineages are found in the Death Valley System, Central Valleys, Lake Lahontan System and Northwest Lakes Great Basin sub-regions of Houghton (1976). *Siphateles mohavensis* are found naturally in the Mojave River basin while *S. snyderi* is found in the Owens Valley. As previously indicated by microsatellite analysis, the ‘toikona’ lineage is distinctive and restricted to the Owens Valley (Chen et al. 2007). With our broad geographic and lineage sampling we identify that the ‘toikona’ Tui Chub has close affinities to other Tui Chubs from the Owens Valley and may be considered a subspecies of *S. snyderi*, but without a formal taxonomic name yet. We represented *S. obesus* with numerous sampling locations and address the composition of this lineage in a separate following section.

The three main lineages of this grouping, *S. mohavensis*, *S. snyderi* and *S. obesus* exhibit substantial mitonuclear discordance (Figs 1, 3). The mitochondrial data from *S. mohavensis* indicates it is the earliest branching lineage of *Siphateles* as defined here, however the nuclear data places *S. mohavensis* as clearly most closely related to *S. snyderi* and *S. obesus*. Geologic evidence indicates that the Owens Valley was the likely source of Mojave River *Siphateles* (Soltz and Naiman 1978) and that there were connections via the Amargosa River to the Mojave River where *S. mohavensis* was naturally distributed. Connections from the Owens Valley to Death Valley did occur up until the end of the Pleistocene, but how much gene flow occurred is unclear. No *Siphateles* are known from recent times in the Amargosa River or other intermediate systems between the Mojave River and Owens River, and if present, would be insightful. In Fig. 1 the branching order of mtDNA lineages may not reflect the true species history and more complex scenarios are not necessary to invoke. The mitonuclear discordance observed between *S. snyderi* and *S. obesus* mtDNA and genome-wide SNP data, however, may require a more complex scenario.

Previously an Owens River fish was sequenced for mitochondrial *cytb* (AF370056.1), and the three Northeast Pond sampling location fish we sequenced shared an identical haplotype. The four other individuals successfully sequenced from *S. snyderi* including toikona lineage fish, only differed by 2–4 mutational steps from each other, but were not all identical. Because these sequenced individuals come from refugial populations that are managed for conservation purposes, there were concerns about the level of potential hybridization in individuals used in translocations into the Owens Valley. However, these *S. snyderi* fish did not show evidence of recent hybridization in the nuclear genome (Fig. 5). While we observe deep divergences in the nuclear data-based analyses, the mitochondrial data from the Owens Valley is nested within a broadly distributed clade of *S. obesus* fish. There are three resultant hypotheses that can be explored. The first is that very recent introgression has occurred leading to the observed patterns. This is unlikely given that there is mitochondrial diversity across the Owens Valley fish sequenced and that all fish sequenced successfully for *cytb* have an *obesus*-like mtDNA lineage. The second hypothesis may be that there was historic gene flow between the Lahontan Basin and the prehistoric Lake Russell, represented today by Mono Lake. There is no current paleo-hydrological information supporting a connection between the Lahontan and Mono Basins after the 3.2 mya closure of the northern outlet of Lake Russell. The presence of active normal and transtensional faulting along the northern edge of the Mono Basin makes the probability of drainage capture a likely mechanism for the introduction of the *S. obesus* mitochondrial lineage into Lake Russell. Subsequent outflow events from Lake Russell would have led to the exchange of mitochondrial DNA lineages between the Lahontan Basin and the Owens Valley, with selection driving the introgression of mtDNA. Such events have been observed in several fish genera leading to examples of mitonuclear discordance (Moyle and Campbell 2022; Campbell et al. 2024). Finally, it is also possible that the depicted pattern cannot be fully interpreted given the few sequences generated. Our effort to generate and sequence *cytb* amplicons from *S. snyderi* was not broadly successful. We initially targeted 12 individuals but generated only seven sequences. Additional sequencing of mitochondrial data from *S. snyderi* and dedicated investigation of *S. mohavensis*, *S. snyderi* and *S. obesus* could be undertaken to clarify the source of this mitonuclear discordance.

### ﻿Composition of *Siphateles obesus*

*Siphateles obesus* is found across the Lake Lahontan System with some locations in the Central Basins and mtDNA sequences from a region of the Northwest Lakes are placed with *S. obesus* though not sequenced from the nuclear genome in this study. There are three main lineages present within *S. obesus* in genomic sequence data examined in this study. The first division is a Fish Lake Valley lineage represented by two sampling locations (Lida Pond and McNett Ranch), with Railroad and Little Fish Lake valleys forming a second lineage, and the remainder of *S. obesus* sampling locations (Walker, Lahontan drainages, etc.) forming a third lineage. The mtDNA data analyzed also contained a distinctive clade from the Summer Lake Basin in Oregon, part of pluvial Lake Chewaucan (Suppl. material 1: fig S1). This biogeographic region was not sampled in this study for genomic sequencing but may be reasoned to be another distinctive genetic lineage within *S. obesus*. This finding presents a disjunct distribution of *S. obesus* that may represent historical processes such as fault-block topography or climatic change resulting in a vicariant event (Harris 2000). Alternatively, the mtDNA may fail to accurately portray evolutionary relationships (e.g., Zink and Barrowclough 2008; Edwards and Bensch 2009).

The Fish Lake Valley lineage is well-supported in phylogenetic analysis, with maximal support for monophyly in concatenated ML and species-tree analyses (Figs 3, 4). Furthermore, it is divided into two discrete units comprising the separate sampling locations (Fig. 3, Suppl. material 1: fig S2). Based on the results of the analyses presented here, it is unlikely that the Lida Pond location outside Fish Lake Valley was founded by human-mediated movement from the McNett Ranch sampling location. The Lida Pond fish appear to have unique genetic diversity within FLVTC not represented in McNett Ranch and that may now be lost from the native range. Resolution of FLVTC as the earliest-branching lineage of *S. obesus* is indicated by our species-tree analysis, though this result receives low (BS = 51%) support (Fig. 4). Similarly in Relict Dace *Relictus solitarius* Hubbs & Miller, 1972, there is clear evidence of separation of populations of this fish in Goshute, Steptoe, and Spring valleys in eastern Nevada from the western populations of this fish. Support for monophyly of eastern valleys is high, but support for branching relationships within the eastern clade are only moderate (BS = 75–78%) in a species tree analysis. Relatively rapid splitting of populations, bottlenecking and genetic drift can act to reduce the number of sites that exist to support branching patterns, producing a lower signal to noise relationship in these desert fishes (Finger et al. 2022).

Geographic evidence supports a migration pathway from the Lahontan Basin into Fish Lake Valley that was disrupted ~2 million years ago by action along the Huntoon Valley fault system (Reheis et al. 2002). After this time, a pluvial lake existed until ~ 0.5 million years with periodic outflows that may have allowed gene flow (Reheis et al. 1991). The phylogenetic placement of FLVTC as closely related to Lahontan lineages with substantial divergence is concordant with this geographical information.

The Railroad Valley and Little Fish Lake Valley lineage also is highly supported (e.g., Fig. 4); however, the separation of Railroad Valley sampling locations at an individual level does not occur (Suppl. material 1: fig S2). This indicates a lack of genetic structuring between Railroad Valley sampling locations. Finally, a third lineage of broadly Lahontan Basin *S. obesus* is present across analyses composed of sampling locations that exhibit genetic structuring (e.g., Figs 3–5).

### ﻿The freshwater biodiversity crisis: a perspective from the Great Basin and adjacent areas

The Great Basin is a relatively accessible area in a developed country, with a limited ichthyofauna. As a result, leuciscid minnows have received substantial anatomical analysis leading to various classification schemes discussed in detail by Simons and Mayden (1998). Importantly, work by Uyeno (1961) lead to the consolidation of numerous fishes under the genus *Gila*, with a reality, as interpreted through molecular phylogenetics, that is much more complex (e.g., Simons and Mayden 1998; Schonhuth et al. 2012). Indeed, the anatomical differentiation of species within *Siphateles* may be challenging as well, and *Siphateles mohavensis*, was noted by Miller (1973: 8) to be a subspecies as *Gila bicolor mohavensis*, accompanied by the statement “I have not been able to discover characters that will separate it specifically from all populations of *Gila bicolor* in the Lahontan basin”. The great amount of anatomical variation across the range of a fish species in the Great Basin, such as all *Siphateles obesus* in the Lahontan Basin, is likely a result of ecological plasticity and the occupation of a diversity of habitats and ecologies. Habitats in the Great Basin include hot springs, cold springs, lakes, and rivers, all with co-occurring food items and other fishes that also vary over such large geographic areas.

*Siphateles mohavensis* is clearly differentiated from other *Siphateles* at mitochondrial and nuclear loci with an ancient movement into the Mojave River and extinction from other habitats that may have been occupied. Molecular phylogenetic analyses as presented here are useful to identify areas where taxonomic refinement is possible. Our work supports the recognition of seven species previously classified under one, as valid. As species are a currency in biodiversity research, lack of recognition of species-level diversity is detrimental to successful conservation actions (Brown and Lomolino 1998). Similar studies have improved taxonomy at the species-level within *Pantosteus* Cope, 1875 and *Rhinichthys* Agassiz, 1849 in the Great Basin and adjacent regions (Unmack et al. 2014; Su et al. 2022; Moyle et al. 2023). Molecular analyses have also indicated that in western North America adjacent to and in the Great Basin *Prosopium williamsoni* (Girard, 1856) and *Catostomus ardens* Jordan & Gilbert, 1881 contain divergent and unrecognized species lineages (Miller 2003; Mock et al. 2006).

Further investigations are warranted to examine these fishes for the presence or not of anatomical characteristics or other traits in support of species descriptions. At a higher level, molecular phylogenetic studies of Great Basin fishes such as this one provide additional taxonomic improvements at the genus-level. Genomic studies examining other taxa such as the Catostomidae may also be important for understanding the generic relationships of that group (e.g., Campbell et al. 2023; Harris et al. 2025). With due consideration and application of integrative approaches (Ottoni et al. 2025), the contribution of the Great Basin to North American ichthyofauna is likely to continue to increase, more accurately informing biodiversity conservation in the region.

In response to the worldwide biodiversity crisis, in species rich systems conservationists advocate the conservation of ‘type locality hot spots’ where there are type localities of multiple and distinct taxonomic groups, such as plant or butterfly species, for example. For freshwater fish, such locations might be in the species-rich Amazon basin (e.g., Azevedo-Santos and Ottoni 2025). However, this conservation strategy falls short in the Great Basin, where the disconnected inland waters are often not species rich, and therefore type localities with only a single species warrant protection. Indeed, some endemic Great Basin fishes like the Relict Dace are the only naturally-distributed fishes found in their habitats even if over a rather large area. In other Great Basin fishes, the type locality may represent the only extant population such as the Wall Canyon Sucker *Catostomus murivallis* Harris, Markle & Campbell, 2025.

While some Great Basin fish species like Relict Dace and the Wall Canyon Sucker still exist in their type localities, many others do not, often due to the introduction of non-native fishes (Cucherousset and Olden 2011) and human alterations to habitats (Ottoni et al. 2023). For example, *S. isolatus* is no longer found in its type locality and *S. mohavensis* was extirpated from its original distribution because of non-native fish introductions (Harris 2000). Another example is the ’toikona’ Tui Chub which was first documented in 1987 from a single location and fish examined in this study descend from 24 fish translocated to artificial refuge pounds in 1989 (Chen et al. 2007). Conservation actions aimed at the type locality of a hypothetical ‘toikona’ Tui Chub taxon would not provide broad conservation benefits to freshwater fishes in the Great Basin. However, integration across aquatic taxa (e.g., including spring snails Gastropoda: Sorbeoconcha: Hydrobiidae) may be an effective conservation strategy that identifies aquatic type locality hotspots that may be emphasized within conservation frameworks in the Great Basin.

## ﻿Conclusions

We find the fishes previously classified under *Siphateles* to merit taxonomic refinement. At the highest level, a deep genetic divergence between the Alvord Basin leuciscids and other fishes is present and we propose *Epizon* gen. nov. for the Alvord Basin leuciscids. Furthermore, what has been consolidated under *S. bicolor* is better characterized as seven species-level entities, all with existing names. Within *S. obesus* we find that Fish Lake Valley Tui Chub is phylogenetically the earliest-branching lineage and is highly genetically differentiated from other *S. obesus* lineages. It also occupies a limited geographic area, separate from all other Tui Chubs. Based on the level of genetic differentiation and geographic distribution of Fish Lake Valley Tui Chub, it merits recognition as a subspecies. Our search through the literature did not find an available name, therefore formal taxonomic description would be necessary to provide an official name for this subspecies.

## Supplementary Material

XML Treatment for Epizon

## ﻿Additional information

### Use of AI

No use of AI was reported.

### Author ORCIDs

Matthew A. Campbell https://orcid.org/0000-0002-5826-0329

Grace Auringer https://orcid.org/0000-0002-7639-3766

Amanda J. Finger https://orcid.org/0000-0003-3850-3685

### Data availability

Demultiplexed NGS sequence data has been deposited to the NCBI Sequence Read Archive under BioProject PRJNA1327467. Sanger sequenced mitochondrial sequences are available from GenBank (https://www.ncbi.nlm.nih.gov/genbank/) with accessions reported in Suppl. material 2. Tree files used in generation of figures and ASAP results are provided in Suppl. material 3. Individual metadata is provided as a Suppl. material 4.

## Supplementary materials

Supplementary material 1 Phylogenetic trees Data type pdf https://binary.pensoft.net/file/1472634This dataset is made available under the Open Database License (http://opendatacommons.org/licenses/odbl/1.0/). The Open Database License (ODbL) is a license agreement intended to allow users to freely share, modify, and use this Dataset while maintaining this same freedom for others, provided that the original source and author(s) are credited.Matthew A. Campbell, Serra C. Perry, Khyana N. Yearwood, Grace Auringer, Nick Buckmaster, Amanda J. Finger

Supplementary material 2 Mitochondrial (cytb) sequences Data type docx Explanation note Mitochondrial cytochrome b (cytb) sequences in species delimitation and phylogenetic analysis. https://binary.pensoft.net/file/1472635This dataset is made available under the Open Database License (http://opendatacommons.org/licenses/odbl/1.0/). The Open Database License (ODbL) is a license agreement intended to allow users to freely share, modify, and use this Dataset while maintaining this same freedom for others, provided that the original source and author(s) are credited.Matthew A. Campbell, Serra C. Perry, Khyana N. Yearwood, Grace Auringer, Nick Buckmaster, Amanda J. Finger

Supplementary material 3 Tree files and ASAP results Data type zip Explanation note Tree files from concatenated and species-tree analyses and ASAP results. https://binary.pensoft.net/file/1472636This dataset is made available under the Open Database License (http://opendatacommons.org/licenses/odbl/1.0/). The Open Database License (ODbL) is a license agreement intended to allow users to freely share, modify, and use this Dataset while maintaining this same freedom for others, provided that the original source and author(s) are credited.Matthew A. Campbell, Serra C. Perry, Khyana N. Yearwood, Grace Auringer, Nick Buckmaster, Amanda J. Finger

Supplementary material 4 Individual metadata Data type csv Explanation note Metadata from the 438 sequences analyzed in this study. TipLabel may be used to rename phylogenetic tree labels, names for samples used in the Genomic Variation Laboratory are included, with geographic information where samples were obtained. The basic taxonomy is given, the number of reads obtained from sequencing, the number of reads after filtering for proper pairing and removal of PCR duplicates ('Dedup'). https://binary.pensoft.net/file/1472637This dataset is made available under the Open Database License (http://opendatacommons.org/licenses/odbl/1.0/). The Open Database License (ODbL) is a license agreement intended to allow users to freely share, modify, and use this Dataset while maintaining this same freedom for others, provided that the original source and author(s) are credited.Matthew A. Campbell, Serra C. Perry, Khyana N. Yearwood, Grace Auringer, Nick Buckmaster, Amanda J. Finger
