# Supplementary material for: ﻿Evolutionary relationships of Fish Lake Valley Tui Chub Siphateles obesus ssp. (Teleostei, Cypriniformes, Leuciscidae) and a new genus of leuciscid minnows from the Alvord Basin, western United States
Source: Zookeys. 2025 Nov 21;1261:39–67. doi: 10.3897/zookeys.1261.151636 (PMC12663728; doi:10.3897/zookeys.1261.151636)
Supplement: Supplementary material 1 — Phylogenetic trees [file zookeys-1261-039_article-151636__-s001.pdf]

Supplementary Figure S1
Maximum Likelihood phylogeny of mitochondrial cytochrome (cytb) data featuring 'Siphateles bicolor'. Tip labels are GenBank accession numbers for the sequences with results of species delimitation of 'S. bicolor' indicated for six clusters. For 'S. bicolor' labeled sequences, geographic information is provided. Nodal support is indicated by gray circles for bootstrap support (BS) > 90%, white circles for 90% > BS > 75% and not indicated for BS < 75%.

Supplementary Figure S2
Maximum Likelihood phylogeny of Siphateles generated from 2,649 SNPs considered variable by IQ-TREE2 with individual sampling locations indicated at tips. Nodal support is indicated by gray circles for bootstrap support (BS) > 90%, white circles for 90% > BS > 75% and not indicated for BS < 75%. **A** contains S. snyderi, S. mohavensis and S. obesus. **B** contains outgroups, Epizon alvordensis, S. isolatus, S. newarkensis, S. thalassinus, and S. bicolor.

Figure S1

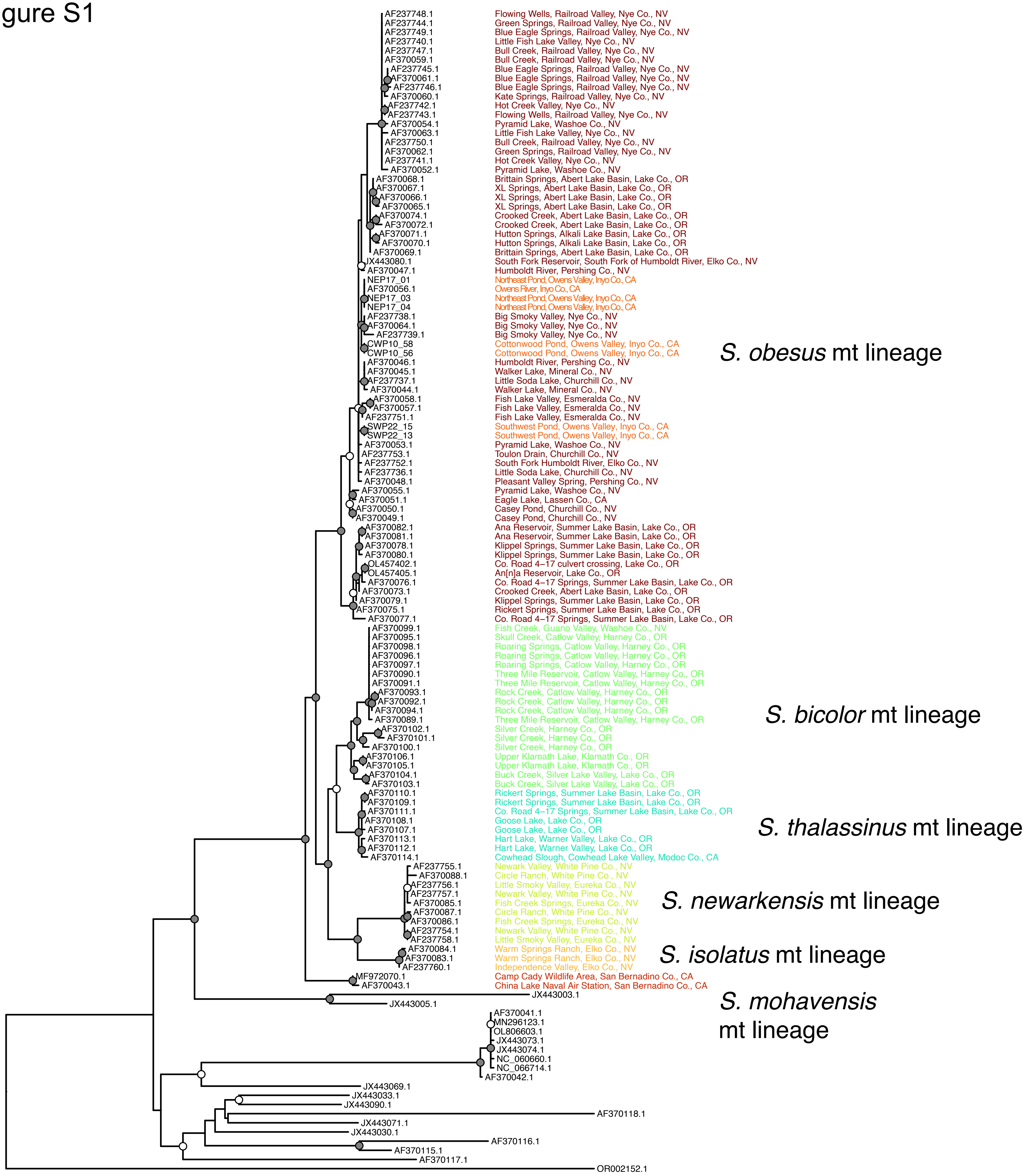
AF237748.1 Flowing Wells, Railroad Valley, Nye Co., NV
AF237744.1 Green Springs, Railroad Valley, Nye Co., NV
AF237749.1 Blue Eagle Springs, Railroad Valley, Nye Co., NV
AF237740.1 Little Fish Lake Valley, Nye Co., NV
AF237747.1 Bull Creek, Railroad Valley, Nye Co., NV
AF370059.1 Bull Creek, Railroad Valley, Nye Co., NV
AF237745.1 Blue Eagle Springs, Railroad Valley, Nye Co., NV
AF370061.1 Blue Eagle Springs, Railroad Valley, Nye Co., NV
AF237746.1 Blue Eagle Springs, Railroad Valley, Nye Co., NV
AF370060.1 Kate Springs, Railroad Valley, Nye Co., NV
AF237742.1 Hot Creek Valley, Nye Co., NV
AF237743.1 Flowing Wells, Railroad Valley, Nye Co., NV
AF370054.1 Pyramid Lake, Washoe Co., NV
AF370063.1 Little Fish Lake Valley, Nye Co., NV
AF237750.1 Bull Creek, Railroad Valley, Nye Co., NV
AF370062.1 Green Springs, Railroad Valley, Nye Co., NV
AF237741.1 Hot Creek Valley, Nye Co., NV
AF370052.1 Pyramid Lake, Washoe Co., NV
AF370068.1 Brittain Springs, Abert Lake Basin, Lake Co., OR
AF370067.1 XL Springs, Abert Lake Basin, Lake Co., OR
AF370066.1 XL Springs, Abert Lake Basin, Lake Co., OR
AF370065.1 XL Springs, Abert Lake Basin, Lake Co., OR
AF370074.1 Crooked Creek, Abert Lake Basin, Lake Co., OR
AF370072.1 Crooked Creek, Abert Lake Basin, Lake Co., OR
AF370071.1 Hutton Springs, Alkali Lake Basin, Lake Co., OR
AF370070.1 Hutton Springs, Alkali Lake Basin, Lake Co., OR
AF370069.1 Brittain Springs, Abert Lake Basin, Lake Co., OR
JX443080.1 South Fork Reservoir, South Fork of Humboldt River, Elko Co., NV
AF370047.1 Humboldt River, Pershing Co., NV
NEP17_01 Northeast Pond, Owens Valley, Inyo Co., CA
AF370056.1 Owens River, Inyo Co., CA
NEP17_03 Northeast Pond, Owens Valley, Inyo Co., CA
NEP17_04 Northeast Pond, Owens Valley, Inyo Co., CA
AF237738.1 Big Smoky Valley, Nye Co., NV
AF370064.1 Big Smoky Valley, Nye Co., NV
AF237739.1 Big Smoky Valley, Nye Co., NV
CWP10_58 Cottonwood Pond, Owens Valley, Inyo Co., CA
CWP10_56 Cottonwood Pond, Owens Valley, Inyo Co., CA
AF370046.1 Humboldt River, Pershing Co., NV
AF370045.1 Walker Lake, Mineral Co., NV
AF237737.1 Little Soda Lake, Churchill Co., NV
AF370044.1 Walker Lake, Mineral Co., NV
AF370058.1 Fish Lake Valley, Esmeralda Co., NV
AF370057.1 Fish Lake Valley, Esmeralda Co., NV
AF237751.1 Fish Lake Valley, Esmeralda Co., NV
SWP22_15 Southwest Pond, Owens Valley, Inyo Co., CA
SWP22_13 Southwest Pond, Owens Valley, Inyo Co., CA
AF370053.1 Pyramid Lake, Washoe Co., NV
AF237753.1 Toulon Drain, Churchill Co., NV
AF237752.1 South Fork Humboldt River, Elko Co., NV
AF237736.1 Little Soda Lake, Churchill Co., NV
AF370048.1 Pleasant Valley Spring, Pershing Co., NV
AF370055.1 Pyramid Lake, Washoe Co., NV
AF370051.1 Eagle Lake, Lassen Co., CA
AF370050.1 Casey Pond, Churchill Co., NV
AF370049.1 Casey Pond, Churchill Co., NV
AF370082.1 Ana Reservoir, Summer Lake Basin, Lake Co., OR
AF370081.1 Ana Reservoir, Summer Lake Basin, Lake Co., OR
AF370078.1 Klippel Springs, Summer Lake Basin, Lake Co., OR
AF370080.1 Klippel Springs, Summer Lake Basin, Lake Co., OR
OL457402.1 Co. Road 4–17 culvert crossing, Lake Co., OR
OL457405.1 An[n]a Reservoir, Lake Co., OR
AF370076.1 Co. Road 4–17 Springs, Summer Lake Basin, Lake Co., OR
AF370073.1 Crooked Creek, Abert Lake Basin, Lake Co., OR
AF370079.1 Klippel Springs, Summer Lake Basin, Lake Co., OR
AF370075.1 Rickert Springs, Summer Lake Basin, Lake Co., OR
AF370077.1 Co. Road 4–17 Springs, Summer Lake Basin, Lake Co., OR
AF370099.1 Fish Creek, Guano Valley, Washoe Co., NV
AF370095.1 Skull Creek, Catlow Valley, Harney Co., OR
AF370098.1 Roaring Springs, Catlow Valley, Harney Co., OR
AF370096.1 Roaring Springs, Catlow Valley, Harney Co., OR
AF370097.1 Roaring Springs, Catlow Valley, Harney Co., OR
AF370090.1 Three Mile Reservoir, Catlow Valley, Harney Co., OR
AF370091.1 Three Mile Reservoir, Catlow Valley, Harney Co., OR
AF370093.1 Rock Creek, Catlow Valley, Harney Co., OR
AF370092.1 Rock Creek, Catlow Valley, Harney Co., OR
AF370094.1 Rock Creek, Catlow Valley, Harney Co., OR
AF370089.1 Three Mile Reservoir, Catlow Valley, Harney Co., OR
AF370102.1 Silver Creek, Harney Co., OR
AF370101.1 Silver Creek, Harney Co., OR
AF370100.1 Silver Creek, Harney Co., OR
AF370106.1 Upper Klamath Lake, Klamath Co., OR
AF370105.1 Upper Klamath Lake, Klamath Co., OR
AF370104.1 Buck Creek, Silver Lake Valley, Lake Co., OR
AF370103.1 Buck Creek, Silver Lake Valley, Lake Co., OR
AF370110.1 Rickert Springs, Summer Lake Basin, Lake Co., OR
AF370109.1 Rickert Springs, Summer Lake Basin, Lake Co., OR
AF370111.1 Co. Road 4–17 Springs, Summer Lake Basin, Lake Co., OR
AF370108.1 Goose Lake, Lake Co., OR
AF370107.1 Goose Lake, Lake Co., OR
AF370113.1 Hart Lake, Warner Valley, Lake Co., OR
AF370112.1 Hart Lake, Warner Valley, Lake Co., OR
AF370114.1 Cowhead Slough, Cowhead Lake Valley, Modoc Co., CA
AF237755.1 Newark Valley, White Pine Co., NV
AF370088.1 Circle Ranch, White Pine Co., NV
AF237756.1 Little Smoky Valley, Eureka Co., NV
AF237757.1 Newark Valley, White Pine Co., NV
AF370085.1 Fish Creek Springs, Eureka Co., NV
AF370087.1 Circle Ranch, White Pine Co., NV
AF370086.1 Fish Creek Springs, Eureka Co., NV
AF237754.1 Newark Valley, White Pine Co., NV
AF237758.1 Little Smoky Valley, Eureka Co., NV
AF370084.1 Warm Springs Ranch, Elko Co., NV
AF370083.1 Warm Springs Ranch, Elko Co., NV
AF237760.1 Independence Valley, Elko Co., NV
MF972070.1 Camp Cady Wildlife Area, San Bernadino Co., CA
AF370043.1 China Lake Naval Air Station, San Bernadino Co., CA
JX443003.1
JX443005.1
AF370041.1
MN296123.1
OL806603.1
JX443073.1
JX443074.1
NC_060660.1
NC_066714.1
AF370042.1
JX443069.1
JX443033.1
JX443090.1
AF370118.1
JX443071.1
JX443030.1
AF370116.1
AF370115.1
AF370117.1
OR002152.1
S. obesus mt lineage
S. bicolor mt lineage
S. thalassinus mt lineage
S. newarkensis mt lineage
S. isolatus mt lineage
S. mohavensis mt lineage

Figure S2

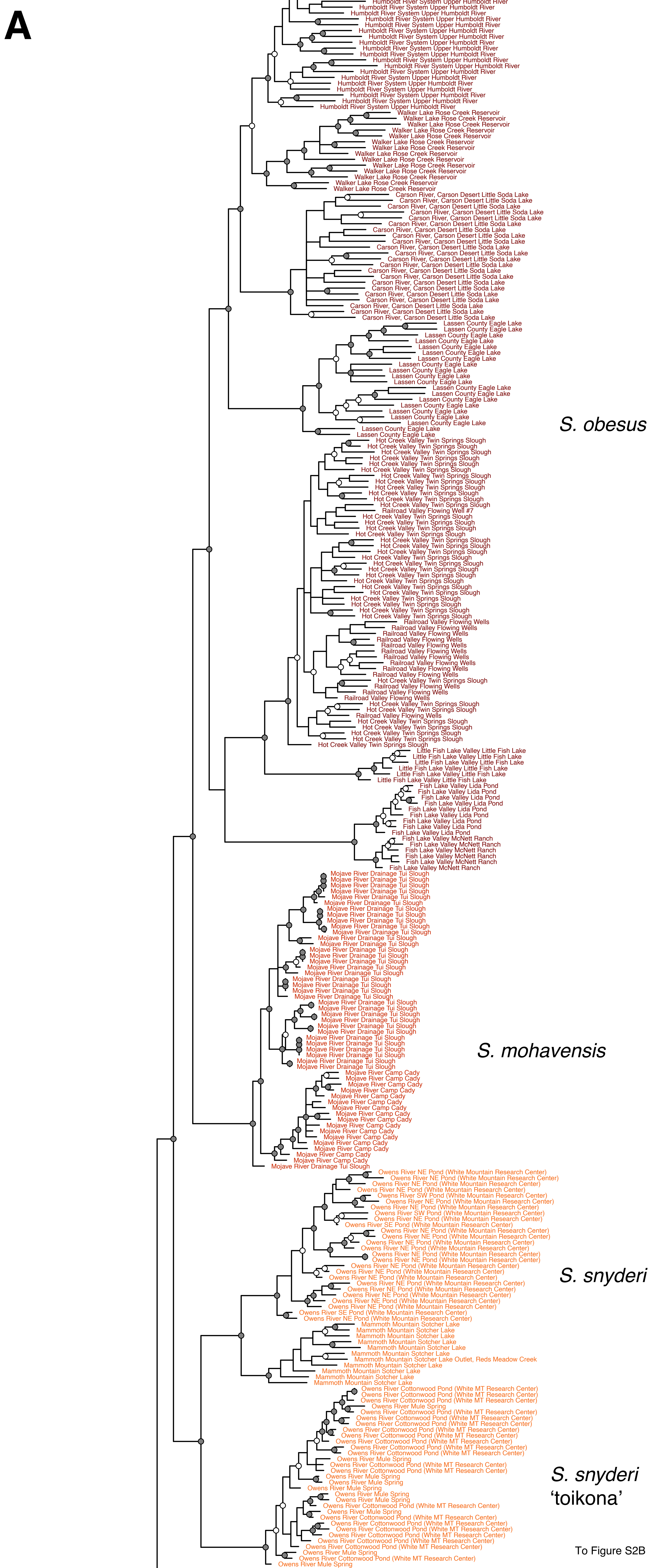
A
Humboldt River System Upper Humboldt River
Humboldt River System Upper Humboldt River
Humboldt River System Upper Humboldt River
Humboldt River System Upper Humboldt River
Humboldt River System Upper Humboldt River
Humboldt River System Upper Humboldt River
Humboldt River System Upper Humboldt River
Humboldt River System Upper Humboldt River
Humboldt River System Upper Humboldt River
Humboldt River System Upper Humboldt River
Humboldt River System Upper Humboldt River
Humboldt River System Upper Humboldt River
Humboldt River System Upper Humboldt River
Humboldt River System Upper Humboldt River
Humboldt River System Upper Humboldt River
Humboldt River System Upper Humboldt River
Humboldt River System Upper Humboldt River
Humboldt River System Upper Humboldt River
Humboldt River System Upper Humboldt River
Humboldt River System Upper Humboldt River
Humboldt River System Upper Humboldt River
Humboldt River System Upper Humboldt River
Humboldt River System Upper Humboldt River
Humboldt River System Upper Humboldt River
Humboldt River System Upper Humboldt River
Walker Lake Rose Creek Reservoir
Walker Lake Rose Creek Reservoir
Walker Lake Rose Creek Reservoir
Walker Lake Rose Creek Reservoir
Walker Lake Rose Creek Reservoir
Walker Lake Rose Creek Reservoir
Walker Lake Rose Creek Reservoir
Walker Lake Rose Creek Reservoir
Walker Lake Rose Creek Reservoir
Walker Lake Rose Creek Reservoir
Walker Lake Rose Creek Reservoir
Walker Lake Rose Creek Reservoir
Walker Lake Rose Creek Reservoir
Walker Lake Rose Creek Reservoir
Carson River, Carson Desert Little Soda Lake
Carson River, Carson Desert Little Soda Lake
Carson River, Carson Desert Little Soda Lake
Carson River, Carson Desert Little Soda Lake
Carson River, Carson Desert Little Soda Lake
Carson River, Carson Desert Little Soda Lake
Carson River, Carson Desert Little Soda Lake
Carson River, Carson Desert Little Soda Lake
Carson River, Carson Desert Little Soda Lake
Carson River, Carson Desert Little Soda Lake
Carson River, Carson Desert Little Soda Lake
Carson River, Carson Desert Little Soda Lake
Carson River, Carson Desert Little Soda Lake
Carson River, Carson Desert Little Soda Lake
Carson River, Carson Desert Little Soda Lake
Carson River, Carson Desert Little Soda Lake
Carson River, Carson Desert Little Soda Lake
Carson River, Carson Desert Little Soda Lake
Carson River, Carson Desert Little Soda Lake
Carson River, Carson Desert Little Soda Lake
Carson River, Carson Desert Little Soda Lake
Carson River, Carson Desert Little Soda Lake
Lassen County Eagle Lake
Lassen County Eagle Lake
Lassen County Eagle Lake
Lassen County Eagle Lake
Lassen County Eagle Lake
Lassen County Eagle Lake
Lassen County Eagle Lake
Lassen County Eagle Lake
Lassen County Eagle Lake
Lassen County Eagle Lake
Lassen County Eagle Lake
Lassen County Eagle Lake
Lassen County Eagle Lake
Lassen County Eagle Lake
Lassen County Eagle Lake
Lassen County Eagle Lake
Lassen County Eagle Lake
Lassen County Eagle Lake
Lassen County Eagle Lake
Lassen County Eagle Lake
S. obesus
Hot Creek Valley Twin Springs Slough
Hot Creek Valley Twin Springs Slough
Hot Creek Valley Twin Springs Slough
Hot Creek Valley Twin Springs Slough
Hot Creek Valley Twin Springs Slough
Hot Creek Valley Twin Springs Slough
Hot Creek Valley Twin Springs Slough
Hot Creek Valley Twin Springs Slough
Hot Creek Valley Twin Springs Slough
Hot Creek Valley Twin Springs Slough
Hot Creek Valley Twin Springs Slough
Hot Creek Valley Twin Springs Slough
Railroad Valley Flowing Well #7
Hot Creek Valley Twin Springs Slough
Hot Creek Valley Twin Springs Slough
Hot Creek Valley Twin Springs Slough
Hot Creek Valley Twin Springs Slough
Hot Creek Valley Twin Springs Slough
Hot Creek Valley Twin Springs Slough
Hot Creek Valley Twin Springs Slough
Hot Creek Valley Twin Springs Slough
Hot Creek Valley Twin Springs Slough
Hot Creek Valley Twin Springs Slough
Hot Creek Valley Twin Springs Slough
Hot Creek Valley Twin Springs Slough
Hot Creek Valley Twin Springs Slough
Hot Creek Valley Twin Springs Slough
Hot Creek Valley Twin Springs Slough
Hot Creek Valley Twin Springs Slough
Hot Creek Valley Twin Springs Slough
Hot Creek Valley Twin Springs Slough
Railroad Valley Flowing Wells
Railroad Valley Flowing Wells
Railroad Valley Flowing Wells
Railroad Valley Flowing Wells
Railroad Valley Flowing Wells
Railroad Valley Flowing Wells
Railroad Valley Flowing Wells
Railroad Valley Flowing Wells
Railroad Valley Flowing Wells
Railroad Valley Flowing Wells
Hot Creek Valley Twin Springs Slough
Railroad Valley Flowing Wells
Railroad Valley Flowing Wells
Railroad Valley Flowing Wells
Hot Creek Valley Twin Springs Slough
Hot Creek Valley Twin Springs Slough
Railroad Valley Flowing Wells
Hot Creek Valley Twin Springs Slough
Hot Creek Valley Twin Springs Slough
Hot Creek Valley Twin Springs Slough
Hot Creek Valley Twin Springs Slough
Hot Creek Valley Twin Springs Slough
Little Fish Lake Valley Little Fish Lake
Little Fish Lake Valley Little Fish Lake
Little Fish Lake Valley Little Fish Lake
Little Fish Lake Valley Little Fish Lake
Little Fish Lake Valley Little Fish Lake
Little Fish Lake Valley Little Fish Lake
Fish Lake Valley Lida Pond
Fish Lake Valley Lida Pond
Fish Lake Valley Lida Pond
Fish Lake Valley Lida Pond
Fish Lake Valley Lida Pond
Fish Lake Valley Lida Pond
Fish Lake Valley Lida Pond
Fish Lake Valley Lida Pond
Fish Lake Valley Lida Pond
Fish Lake Valley McNett Ranch
Fish Lake Valley McNett Ranch
Fish Lake Valley McNett Ranch
Fish Lake Valley McNett Ranch
Fish Lake Valley McNett Ranch
Fish Lake Valley McNett Ranch
Mojave River Drainage Tui Slough
Mojave River Drainage Tui Slough
Mojave River Drainage Tui Slough
Mojave River Drainage Tui Slough
Mojave River Drainage Tui Slough
Mojave River Drainage Tui Slough
Mojave River Drainage Tui Slough
Mojave River Drainage Tui Slough
Mojave River Drainage Tui Slough
Mojave River Drainage Tui Slough
Mojave River Drainage Tui Slough
Mojave River Drainage Tui Slough
Mojave River Drainage Tui Slough
Mojave River Drainage Tui Slough
Mojave River Drainage Tui Slough
Mojave River Drainage Tui Slough
Mojave River Drainage Tui Slough
Mojave River Drainage Tui Slough
Mojave River Drainage Tui Slough
Mojave River Drainage Tui Slough
Mojave River Drainage Tui Slough
Mojave River Drainage Tui Slough
Mojave River Drainage Tui Slough
Mojave River Drainage Tui Slough
Mojave River Drainage Tui Slough
Mojave River Drainage Tui Slough
Mojave River Drainage Tui Slough
Mojave River Drainage Tui Slough
Mojave River Drainage Tui Slough
Mojave River Drainage Tui Slough
Mojave River Drainage Tui Slough
Mojave River Drainage Tui Slough
Mojave River Drainage Tui Slough
Mojave River Drainage Tui Slough
S. mohavensis
Mojave River Camp Cady
Mojave River Camp Cady
Mojave River Camp Cady
Mojave River Camp Cady
Mojave River Camp Cady
Mojave River Camp Cady
Mojave River Camp Cady
Mojave River Camp Cady
Mojave River Camp Cady
Mojave River Camp Cady
Mojave River Camp Cady
Mojave River Camp Cady
Mojave River Camp Cady
Mojave River Camp Cady
Mojave River Camp Cady
Mojave River Camp Cady
Mojave River Drainage Tui Slough
Owens River NE Pond (White Mountain Research Center)
Owens River NE Pond (White Mountain Research Center)
Owens River NE Pond (White Mountain Research Center)
Owens River NE Pond (White Mountain Research Center)
Owens River SW Pond (White Mountain Research Center)
Owens River NE Pond (White Mountain Research Center)
Owens River NE Pond (White Mountain Research Center)
Owens River SW Pond (White Mountain Research Center)
Owens River NE Pond (White Mountain Research Center)
Owens River SE Pond (White Mountain Research Center)
Owens River NE Pond (White Mountain Research Center)
Owens River NE Pond (White Mountain Research Center)
Owens River NE Pond (White Mountain Research Center)
Owens River NE Pond (White Mountain Research Center)
Owens River NE Pond (White Mountain Research Center)
Owens River NE Pond (White Mountain Research Center)
Owens River NE Pond (White Mountain Research Center)
Owens River NE Pond (White Mountain Research Center)
Owens River NE Pond (White Mountain Research Center)
Owens River NE Pond (White Mountain Research Center)
Owens River NE Pond (White Mountain Research Center)
Owens River NE Pond (White Mountain Research Center)
Owens River NE Pond (White Mountain Research Center)
Owens River NE Pond (White Mountain Research Center)
Owens River SE Pond (White Mountain Research Center)
Owens River NE Pond (White Mountain Research Center)
S. snyderi
Mammoth Mountain Sotcher Lake
Mammoth Mountain Sotcher Lake
Mammoth Mountain Sotcher Lake
Mammoth Mountain Sotcher Lake
Mammoth Mountain Sotcher Lake
Mammoth Mountain Sotcher Lake
Mammoth Mountain Sotcher Lake Outlet, Reds Meadow Creek
Mammoth Mountain Sotcher Lake
Mammoth Mountain Sotcher Lake
Mammoth Mountain Sotcher Lake
Mammoth Mountain Sotcher Lake
Owens River Cottonwood Pond (White MT Research Center)
Owens River Cottonwood Pond (White MT Research Center)
Owens River Cottonwood Pond (White MT Research Center)
Owens River Mule Spring
Owens River Cottonwood Pond (White MT Research Center)
Owens River Cottonwood Pond (White MT Research Center)
Owens River Cottonwood Pond (White MT Research Center)
Owens River Cottonwood Pond (White MT Research Center)
Owens River Cottonwood Pond (White MT Research Center)
Owens River Cottonwood Pond (White MT Research Center)
Owens River Cottonwood Pond (White MT Research Center)
Owens River Cottonwood Pond (White MT Research Center)
Owens River Mule Spring
Owens River Cottonwood Pond (White MT Research Center)
Owens River Cottonwood Pond (White MT Research Center)
Owens River Mule Spring
Owens River Mule Spring
Owens River Mule Spring
Owens River Mule Spring
Owens River Mule Spring
Owens River Cottonwood Pond (White MT Research Center)
Owens River Mule Spring
Owens River Cottonwood Pond (White MT Research Center)
Owens River Cottonwood Pond (White MT Research Center)
Owens River Cottonwood Pond (White MT Research Center)
Owens River Cottonwood Pond (White MT Research Center)
Owens River Cottonwood Pond (White MT Research Center)
Owens River Cottonwood Pond (White MT Research Center)
Owens River Mule Spring
Owens River Cottonwood Pond (White MT Research Center)
Owens River Mule Spring
S. snyderi
'toikona'
To Figure S2B

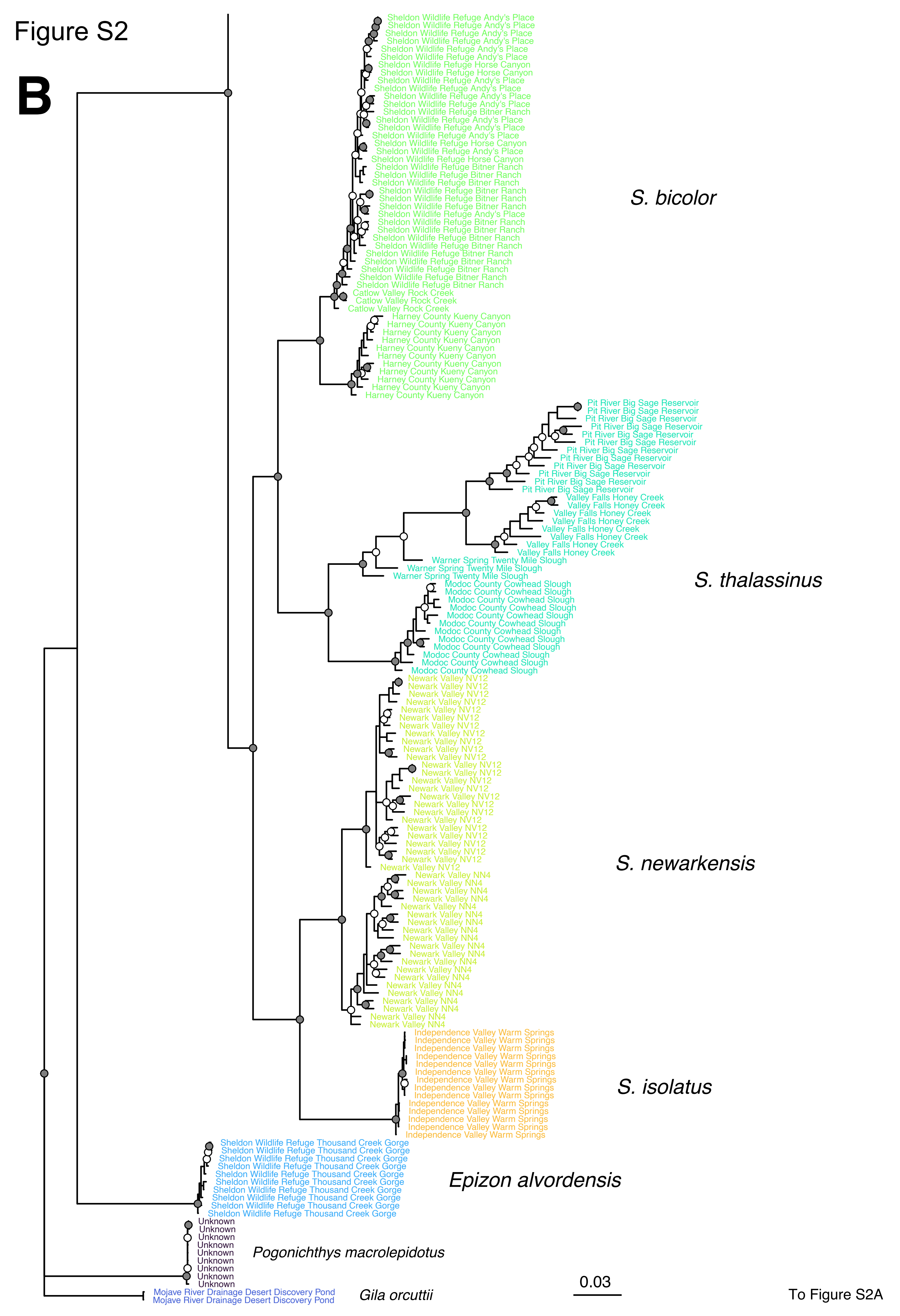

Figure S2
B
S. bicolor
S. thalassinus
S. newarkensis
S. isolatus
Epizon alvordensis
Pogonichthys macrolepidotus
Gila orcuttii
Sheldon Wildlife Refuge Andy's Place
Sheldon Wildlife Refuge Horse Canyon
Sheldon Wildlife Refuge Bitner Ranch
Catlow Valley Rock Creek
Harney County Kueny Canyon
Pit River Big Sage Reservoir
Valley Falls Honey Creek
Warner Spring Twenty Mile Slough
Modoc County Cowhead Slough
Newark Valley NV12
Newark Valley NN4
Independence Valley Warm Springs
Sheldon Wildlife Refuge Thousand Creek Gorge
Unknown
Mojave River Drainage Desert Discovery Pond
0.03
To Figure S2A
