# Supplementary material for: ﻿Evolutionary relationships of Fish Lake Valley Tui Chub Siphateles obesus ssp. (Teleostei, Cypriniformes, Leuciscidae) and a new genus of leuciscid minnows from the Alvord Basin, western United States
Source: Zookeys. 2025 Nov 21;1261:39–67. doi: 10.3897/zookeys.1261.151636 (PMC12663728; doi:10.3897/zookeys.1261.151636)
Supplement: Supplementary material 2 — Mitochondrial (cytb) sequences [file zookeys-1261-039_article-151636__-s002.docx]

Supplementary Table S1

Mitochondrial cytochrome b (*cytb*) sequences in species delimitation and phylogenetic analysis. Accession numbers, classification from the NCBI on GenBank and species assignment from Assemble Species by Automatic Partitioning (ASAP) are presented. Any voucher information associated with the accession is given from the GenBank record of the sequence along with geographic location information Siphateles species included are *S. thalassinus* (Cope, 1883), *S. bicolor* (Girard, 1856), *S. newarkensis* (Hubbs & Miller, 1972), *S. isolatus* (Hubbs & Miller, 1972), *S. snyderi* (Miller, 1973), *S. mohavensis* Snyder, 1918, and *S. obesus* (Girard, 1856). Species of *Epizon* gen. nov. included are *E. alvordensis* (Hubbs & Mlller, 1972) and *E. boraxobius* (Williams & Bond, 1980).

| **Accession** | **NCBI species** | | **NCBI subspecies** | **Species from ASAP analysis** | **Voucher if known** | **Location information** |
| --- | --- | --- | --- | --- | --- | --- |
| Ingroup – *Siphateles* |  | |  |  |  |  |
| AF370043.1 | *bicolor* | | *mohavensis* | *mohavensis* | BB43 | China Lake Naval Air Station, San Bernadino County, California |
| AF370044.1 | *bicolor* | | *obesa* | *obesus* | OS 15111-1 | Walker Lake, Mineral County, Nevada |
| AF370045.1 | *bicolor* | | *obesa* | *obesus* | OS 15111-2 | Walker Lake, Mineral County, Nevada |
| AF370046.1 | *bicolor* | | *obesa* | *obesus* | OS 15637-1 | Humboldt River, Pershing County, Nevada |
| AF370047.1 | *bicolor* | | *obesa* | *obesus* | OS 15637-3 | Humboldt River, Pershing County, Nevada |
| AF370048.1 | *bicolor* | | *obesa* | *obesus* | OS 15512-2 | Pleasant Valley Spring, Pershing County, Nevada |
| AF370049.1 | *bicolor* | | *obesa* | *obesus* | OS 15513 | Casey Pond, Churchill County, Nevada |
| AF370050.1 | *bicolor* | | *obesa* | *obesus* | OS 15513-2 | Casey Pond, Churchill County, Nevada |
| AF370051.1 | *bicolor* | | *obesa* | *obesus* | BB18 | Eagle Lake, Lassen County, California |
| AF370052.1 | *bicolor* | | *obesa* | *obesus* | BB25 | Pyramid Lake, Washoe County, Nevada |
| AF370053.1 | *bicolor* | | *obesa* | *obesus* | BB28 | Pyramid Lake, Washoe County, Nevada |
| AF370054.1 | *bicolor* | | *obesa* | *obesus* | BB48 | Pyramid Lake, Washoe County, Nevada |
| AF370055.1 | *bicolor* | | *obesa* | *obesus* | BB49 | Pyramid Lake, Washoe County, Nevada |
| AF370056.1 | *bicolor* | | *obesa* | *obesus* | OS 15747-1 | Owens River, Inyo County, California |
| AF370057.1 | *bicolor* | | *obesa* | *obesus* | OS 15520-1 | Fish Lake Valley, Esmeralda County, Nevada |
| AF370058.1 | *bicolor* | | *obesa* | *obesus* | OS 15520-2 | Fish Lake Valley, Esmeralda County, Nevada |
| AF370059.1 | *bicolor* | | *obesa* | *obesus* | OS 15514-1 | Bull Creek, Railroad Valley, Nye County, Nevada |
| AF370060.1 | *bicolor* | | *obesa* | *obesus* | OS 15515-2 | Kate Springs, Railroad Valley, Nye County, Nevada |
| AF370061.1 | *bicolor* | | *obesa* | *obesus* | OS 15517-2 | Blue Eagle Springs, Railroad Valley, Nye County, Nevada |
| AF370062.1 | *bicolor* | | *obesa* | *obesus* | OS 15519-2 | Green Springs, Railroad Valley, Nye County, Nevada |
| AF370063.1 | *bicolor* | | *obesa* | *obesus* | BB1 | Little Fish Lake Valley, Nye County, Nevada |
| AF370064.1 | *bicolor* | | *obesa* | *obesus* | OS 15522-1 | Big Smokey Valley, Nye County, Nevada |
| AF370065.1 | *bicolor* | | *obesa* | *obesus* | OS 16774-1 | XL Springs, Abert Lake Basin, Lake County, Oregon |
| AF370066.1 | *bicolor* | | *obesa* | *obesus* | OS 16774-2 | XL Springs, Abert Lake Basin, Lake County, Oregon |
| AF370067.1 | *bicolor* | | *obesa* | *obesus* | OS 16774-3 | XL Springs, Abert Lake Basin, Lake County, Oregon |
| AF370068.1 | *bicolor* | | *obesa* | *obesus* | OS 16722-1 | Brittain Springs, Abert Lake Basin, Lake County, Oregon |
| AF370069.1 | *bicolor* | | *obesa* | *obesus* | OS 16722-2 | Brittain Springs, Abert Lake Basin, Lake County, Oregon |
| AF370070.1 | *bicolor* | | *obesa* | *obesus* | OS 16771-1 | Hutton Springs, Alkali Lake Basin, Lake County, Oregon |
| AF370071.1 | *bicolor* | | *obesa* | *obesus* | OS 16771-3 | Hutton Springs, Alkali Lake Basin, Lake County, Oregon |
| AF370072.1 | *bicolor* | | *obesa* | *obesus* | PMH9503-1 | Crooked Creek, Abert Lake Basin, Lake County, Oregon |
| AF370073.1 | *bicolor* | | *obesa* | *obesus* | PMH9503-2 | Crooked Creek, Abert Lake Basin, Lake County, Oregon |
| AF370074.1 | *bicolor* | | *obesa* | *obesus* | PMH9503-3 | Crooked Creek, Abert Lake Basin, Lake County, Oregon |
| AF370075.1 | *bicolor* | | *obesa* | *obesus* | OS 15312-33 | Rickert Springs, Summer Lake Basin, Lake County, Oregon |
| AF370076.1 | *bicolor* | | *obesa* | *obesus* | OS 15437-47 | County Road 4-17 Springs, Summer Lake Basin, Lake County, Oregon |
| AF370077.1 | *bicolor* | | *obesa* | *obesus* | OS 15437-49 | County Road 4-17 Springs, Summer Lake Basin, Lake County, Oregon |
| AF370078.1 | *bicolor* | | *obesa* | *obesus* | OS 15438-16 | Klippel Springs, Summer Lake Basin, Lake County, Oregon |
| OL457405.1 | *bicolor* | | *obesa* | *obesus* |  | An[n]a Reservoir, Lake County, Oregon |
| OL457402.1 | *bicolor* | | *obesa* | *obesus* |  | County Road 4-17 culvert crossing, Lake County, Oregon |
| JX443080.1 | *bicolor* | | *obesa* | *obesus* | OS 15637-3 | South Fork Reservoir, South Fork of Humboldt River, Elko County, Nevada |
| AF237736.1 | *bicolor* | | *obesa* | *obesus* |  | Little Soda Lake, Churchill County, Nevada |
| AF237737.1 | *bicolor* | | *obesa* | *obesus* |  | Little Soda Lake, Churchill County, Nevada |
| AF237738.1 | *bicolor* | | *obesa* | *obesus* |  | Big Smoky Valley, Nye County, Nevada |
| AF370079.1 | *bicolor* | | *obesa* | *obesus* | OS 15438-17 | Klippel Springs, Summer Lake Basin, Lake County, Oregon |
| AF370080.1 | *bicolor* | | *obesa* | *obesus* | OS 15438-18 | Klippel Springs, Summer Lake Basin, Lake County, Oregon |
| AF370081.1 | *bicolor* | | *obesa* | *obesus* | OS 15440-2 | Ana Reservoir, Summer Lake Basin, Lake County, Oregon |
| AF370082.1 | *bicolor* | | *obesa* | *obesus* | OS 15440-4 | Ana Reservoir, Summer Lake Basin, Lake County, Oregon |
| AF370083.1 | *bicolor* | | *isolata* | *isolatus* | OS 15622-1 | Warm Springs Ranch, Elko County, Nevada |
| AF370084.1 | *bicolor* | | *isolata* | *isolatus* | OS 15622-3 | Warm Springs Ranch, Elko County, Nevada |
| AF370085.1 | *bicolor* | | *newarkensis* | *newarkensis* | OS 15743-1 | Fish Creek Springs, Eureka County, Nevada |
| AF370086.1 | *bicolor* | | *newarkensis* | *newarkensis* | OS 15743-2 | Fish Creek Springs, Eureka County, Nevada |
| AF370087.1 | *bicolor* | | *newarkensis* | *newarkensis* | OS 15744-1 | Circle Ranch, White Pine County, Nevada |
| AF370088.1 | *bicolor* | | *newarkensis* | *newarkensis* | OS 15744-2 | Circle Ranch, White Pine County, Nevada |
| AF370089.1 | *bicolor* | | *eurysoma* | *bicolor* | OS 15572-1 | Three Mile Reservoir, Catlow Valley, Harney County, Oregon |
| AF370090.1 | *bicolor* | | *eurysoma* | *bicolor* | OS 15572-2 | Three Mile Reservoir, Catlow Valley, Harney County, Oregon |
| AF370091.1 | *bicolor* | | *eurysoma* | *bicolor* | OS 15572-3 | Three Mile Reservoir, Catlow Valley, Harney County, Oregon |
| AF370092.1 | *bicolor* | | *eurysoma* | *bicolor* | OS 15635-1 | Rock Creek, Catlow Valley, Harney County, Oregon |
| AF370093.1 | *bicolor* | | *eurysoma* | *bicolor* | OS 15635-2 | Rock Creek, Catlow Valley, Harney County, Oregon |
| AF370094.1 | *bicolor* | | *eurysoma* | *bicolor* | OS 15635-3 | Rock Creek, Catlow Valley, Harney County, Oregon |
| AF370095.1 | *bicolor* | | *eurysoma* | *bicolor* | PMH9504-3 | Skull Creek, Catlow Valley, Harney County, Oregon |
| AF370096.1 | *bicolor* | | *eurysoma* | *bicolor* | PMH9505-1 | Roaring Springs, Catlow Valley, Harney County, Oregon |
| AF370097.1 | *bicolor* | | *eurysoma* | *bicolor* | PMH9505-2 | Roaring Springs, Catlow Valley, Harney County, Oregon |
| AF370098.1 | *bicolor* | | *eurysoma* | *bicolor* | PMH9505-3 | Roaring Springs, Catlow Valley, Harney County, Oregon |
| AF370099.1 | *bicolor* | | *eurysoma* | *bicolor* | OS 15574-4 | Fish Creek, Guano Valley, Washoe County, Nevada |
| AF370100.1 | *bicolor* | | *columbianus* | *bicolor* | OS 15577-4 | Silver Creek, Harney County, Oregon |
| AF370101.1 | *bicolor* | | *columbianus* | *bicolor* | OS 15577-6 | Silver Creek, Harney County, Oregon |
| AF370102.1 | *bicolor* | | *columbianus* | *bicolor* | OS 15577-8 | Silver Creek, Harney County, Oregon |
| AF370103.1 | *bicolor* | | *subsp* | *bicolor* | PMH9401-1 | Buck Creek, Silver Lake Valley, Lake County, Oregon |
| AF370104.1 | *bicolor* | | *subsp* | *bicolor* | PMH9401-2 | Buck Creek, Silver Lake Valley, Lake County, Oregon |
| AF370105.1 | *bicolor* | | *subsp* | *bicolor* | OS 16665-1 | Upper Klamath Lake, Klamath County, Oregon |
| AF370106.1 | *bicolor* | | *subsp* | *bicolor* | OS 16665-2 | Upper Klamath Lake, Klamath County, Oregon |
| AF370107.1 | *bicolor* | | *thalassinus* | *thalassinus* | BB60 | Goose Lake, Lake County, Oregon |
| AF370108.1 | *bicolor* | | *thalassinus* | *thalassinus* | BB62 | Goose Lake, Lake County, Oregon |
| AF370109.1 | *bicolor* | | *thalassinus* | *thalassinus* | OS 15312-32 | Rickert Springs, Summer Lake Basin, Lake County, Oregon |
| AF370110.1 | *bicolor* | | *thalassinus* | *thalassinus* | OS 15312-31 | Rickert Springs, Summer Lake Basin, Lake County, Oregon |
| AF370111.1 | *bicolor* | | *thalassinus* | *thalassinus* | OS 15437-48 | County Road 4-17 Springs, Summer Lake Basin, Lake County, Oregon |
| AF370112.1 | *bicolor* | | *thalassinus* | *thalassinus* | BB-W1 | Hart Lake, Warner Valley, Lake County, Oregon |
| AF370113.1 | *bicolor* | | *thalassinus* | *thalassinus* | BB-W2 | Hart Lake, Warner Valley, Lake County, Oregon |
| AF370114.1 | *bicolor* | | *thalassinus* | *thalassinus* | DFM9709-1 | Cowhead Slough, Cowhead Lake Valley, Modoc County, California |
| MF972070.1 | *bicolor* | | *mohavensis* | *mohavensis* |  | Camp Cady Wildlife Area, San Bernadino County, California |
| AF237760.1 | *bicolor* | | *isolata* | *isolatus* |  | Independence Valley, Elko County, Nevada |
| AF237758.1 | *bicolor* | | *euchila* | *isolatus* |  | Little Smoky Valley, Eureka County, Nevada |
| AF237757.1 | *bicolor* | | *newarkensis* | *newarkensis* |  | Newark Valley, White Pine County, Nevada |
| AF237756.1 | *bicolor* | | *euchila* | *isolatus* |  | Little Smoky Valley, Eureka County, Nevada |
| AF237755.1 | *bicolor* | | *newarkensis* | *newarkensis* |  | Newark Valley, White Pine County, Nevada |
| AF237754.1 | *bicolor* | | *newarkensis* | *newarkensis* |  | Newark Valley, White Pine County, Nevada |
| AF237753.1 | *bicolor* | | *obesa* | *obesus* |  | Toulon Drain, Churchill County, Nevada |
| AF237752.1 | *bicolor* | | *obesa* | *obesus* |  | South Fork Humboldt River, Elko County, Nevada |
| AF237751.1 | *bicolor* | | *obesa* | *obesus* |  | Fish Lake Valley, Esmeralda County, Nevada |
| AF237750.1 | *bicolor* | | *obesa* | *obesus* |  | Bull Creek, Railroad Valley, Nye County, Nevada |
| AF237749.1 | *bicolor* | | *obesa* | *obesus* |  | Blue Eagle Springs, Railroad Valley, Nye County, Nevada |
| AF237748.1 | *bicolor* | | *obesa* | *obesus* |  | Flowing Wells, Railroad Valley, Nye County, Nevada |
| AF237747.1 | *bicolor* | | *obesa* | *obesus* |  | Bull Creek, Railroad Valley, Nye County, Nevada |
| AF237746.1 | *bicolor* | | *obesa* | *obesus* |  | Blue Eagle Springs, Railroad Valley, Nye County, Nevada |
| AF237745.1 | *bicolor* | | *obesa* | *obesus* |  | Blue Eagle Springs, Railroad Valley, Nye County, Nevada |
| AF237744.1 | *bicolor* | | *obesa* | *obesus* |  | Green Springs, Railroad Valley, Nye County, Nevada |
| AF237743.1 | *bicolor* | | *obesa* | *obesus* |  | Flowing Wells, Railroad Valley, Nye County, Nevada |
| AF237742.1 | *bicolor* | | *obesa* | *obesus* |  | Hot Creek Valley, Nye County, Nevada |
| AF237741.1 | *bicolor* | | *obesa* | *obesus* |  | Hot Creek Valley, Nye County, Nevada |
| AF237740.1 | *bicolor* | | *obesa* | *obesus* |  | Little Fish Lake Valley, Nye County, Nevada |
| AF237739.1 | *bicolor* | | *obesa* | *obesus* |  | Big Smoky Valley, Nye County, Nevada |
| PX353425.1 | *bicolor* | | *snyderi* | *obesus* |  | Cottonwood Pond, Owens Valley, Inyo County, California |
| PX353426.1 | *bicolor* | | *snyderi* | *obesus* |  | Cottonwood Pond, Owens Valley, Inyo County, California |
| PX353427.1 | *bicolor* | | *snyderi* | *obesus* |  | Northeast Pond, Owens Valley, Inyo County, California |
| PX353428.1 | *bicolor* | | *snyderi* | *obesus* |  | Northeast Pond, Owens Valley, Inyo County, California |
| PX353429.1 | *bicolor* | | *snyderi* | *obesus* |  | Northeast Pond, Owens Valley, Inyo County, California |
| PX353430.1 | *bicolor* | *snyderi* | | *obesus* |  | Southwest Pond, Owens Valley, Inyo County, California |
| PX353431.1 | *bicolor* | | *snyderi* | *obesus* |  | Southwest Pond, Owens Valley, Inyo County, California |
|  |  | |  |  |  |  |
| *Epizon* gen. nov. |  | |  |  |  |  |
| AF370041 | *alvordensis* | |  |  | PMH9403-1 | Trout Creek, Harney County, Oregon |
| NC_060660.1 | *alvordensis* | |  |  | OS:2011-IC-011-1981 | Serrano Spring, Harney County, Oregon |
| JX443073.1 | *alvordensis* | |  |  |  | Oregon |
| OL806603.1 | *alvordensis* | |  |  |  | Jana Pond close to Borax Lake, Harney County, Oregon |
| AF370042.1 | *boraxobius* | |  |  | PMH9316-10 | Borax Lake, Harney County, Oregon |
| NC_066714.1 | *boraxobius* | |  |  | OS 2009-IC-003 18057 | Borax Lake, Harney County, Oregon |
| MN296123 | *boraxobius* | |  |  | OS:2009-IC-003-18056 | Borax Lake, Harney County, Oregon |
| JX443074.1 | *boraxobius* | |  |  |  | Oregon |
|  |  | |  |  |  |  |
| Outgroups |  | |  |  |  |  |
| OR002152.1 | *Chrosomus erythrogaster* | | (Rafinesque, 1820) |  | KU 43016 | Crooked Creek, Meramec River, Crawford County, Missouri |
| AF370117.1 | *Eremichthys acros* | | Hubbs & Miller, 1948 |  | LVT1537 | Soldier Meadows, Humboldt County, Nevada |
| AF370115.1 | *Relictus solitarius* | | Hubbs & Miller, 1972 |  | OS 15745-1 | Odgers Creek, Ruby Valley, Elko County, Nevada |
| AF370116.1 | *Hesperoleucus symmetricus* | | (Baird & Girard, 1854) |  | OS 15746-1 | Ana River, Summer Lake Basin, Lake County, Oregon |
| JX443030.1 | *Gila coerulea* | | (Girard, 1856) |  | BYU 239498 | Upper Klamath Lake, Oregon |
| AF370118,1 | *Gila orcuttii* | | (Eigenmann & Eigenmann, 1890) |  | OS 15748-1 | Rainbow Creek, San Diego County, California |
| JX443033.1 | *Gila robusta* | | Baird & Girard, 1853 |  |  | Arizona |
| JX443090.1 | *Acrocheilus alutaceus* | | (Agassiz & Pickering, 1855) |  | UAIC 11365.01 | British Columbia, Canada |
| JX443071.1 | *Ptychocheilus lucius* | | Girard, 1856 |  |  | USA |
| JX443069.1 | *Ptychocheilus grandis* | | (Ayres, 1854). |  | UAIC 11548.01 | California |
| JX443003.1 | *Lavinia exilicauda* | | Baird & Girard, 1854 |  | OS 015074 | Putah Creek, University of California Davis, California |
| JX443005.1 | *Mylopharodon conocephalus* | | (Baird & Girard, 1854) |  | UAIC 11548.02 | California |
